# Tianma Granules Alleviate AOM/DSS‐Induced Colorectal Tumorigenesis by Inhibiting the Wnt/β‐Catenin Pathway Activation

**DOI:** 10.1111/jcmm.70772

**Published:** 2025-08-10

**Authors:** Renjing Lin, Jinyin Xiao, Yanjie Chen, Xu Li, Peiwen Zhang, Runcheng Zhang, Min Luo

**Affiliations:** ^1^ Department of Proctology The Second Affiliated Hospital of Hunan University of Chinese Medicine Changsha Hunan China; ^2^ Hunan University of Chinese Medicine Changsha Hunan China

**Keywords:** AOM/DSS, colorectal cancer, inflammation, Tianma granules, Wnt signalling pathway

## Abstract

This study aimed to investigate the anti‐tumour effect and the possible molecular mechanism of Tianma granules on colorectal cancer (CRC). The therapeutic effect of Tianma granules on CRC cell lines (HT116 and SW480) and AOM/DSS‐induced CRC mouse models was evaluated. Tianma granules can attenuate weight loss and increase the survival rate of CRC mice, restore reduced colon length, reduce tumour numbers and increase goblet cell numbers in CRC mice. Tianma granules also downregulated the level of CRC‐specific markers (COX2 and MUC2), inhibited the inflammation (decreased TNF‐α, IL‐1β, IL‐6 levels and increased INF‐γ level), and promoted apoptosis (decreased TUNEL positive cell rate; decreased Bax and Cleaved caspase3 protein levels and increased Bcl2 level) in CRC mice. In vitro, Tianma granules can inhibit the viability, proliferation, migration and invasion of CRC cells, while promoting cell apoptosis, cell cycle arrest and cell senescence. Tianma granules promoted AXIN1 protein levels and inhibited p‐GSK‐3β, β‐catenin, Wnt5a and Cyclin D1 and c‐Myc protein levels. Moreover, the network pharmacology analysis and in vitro validation revealed berberine might be the key compound responsible for Tianma granules' pharmacological actions. In conclusion, Tianma granules can inhibit inflammation and tumour progression in AOM/DSS‐induced CRC mice, as well as inhibit CRC cell malignant phenotype. The protection of Tianma granules against CRC may be achieved by inhibiting the Wnt signalling pathway.

## 1 Introduction

Colorectal cancer (CRC) is a malignant tumour of the digestive system. It is the third most common cancer worldwide, with the incidence and mortality rates second only to gastric and liver cancer [1, 2]. Over the past 30 years, CRC has experienced an increasing incidence rate in China, accompanied by a continuous upward trend [3]. According to China's official statistics for 2020, CRC ranked second in terms of the highest incidence rate and fourth in terms of the highest mortality rate among all malignant tumours [3]. Current CRC clinical treatments primarily rely on surgery, supplemented with chemotherapy, radiotherapy and targeted therapy; these therapies can effectively prolong the survival of CRC patients [4]. However, these treatment regimens entail potential side effects. For example, although 5‐fluorouracil (5‐Fu) functions as the first‐line basic chemotherapy drug for CRC, it may also entail side effects such as drug resistance [5]. It has been shown that in clinical practice, using different drugs to treat cancer concomitantly can reduce the dosage of a single drug, provide synergistic effects and alleviate drug toxicity and side effects [6]. Therefore, the exploration of potential drugs for the synergistic treatment of CRC is amply warranted.

Growing evidence suggests that chronic intestinal inflammation is intricately linked to the pathogenesis of CRC [7, 8]. It has been previously concluded that inflammatory bowel disease (IBD), the chronic inflammation of the intestine, contributes to increased CRC risk [9]. Clinical research has evidenced that nonsteroidal anti‐inflammatory drugs can protect IBD patients from CRC exacerbation [10]. In particular, aspirin can effectively improve the overall survival of CRC patients [11]. However, long‐term use of nonsteroidal anti‐inflammatory drugs will increase the risk of intestinal, renal and cardiovascular complications [12]. Therefore, the exploration of novel drugs with low side effects and low drug tolerance for IBD prevention and treatment is amply warranted, thereby reducing the risk of IBD progressing into CRC.

Traditional Chinese medicine (TCM) has been characterised by its high therapeutic activity and low toxic side effects and has recently garnered academic attention. Multiple studies have provided evidence that TCM can alleviate CRC. For example, Gegen Qinlian decoction can enhance immunity and protect intestinal barrier function in CRC patients [13]. Radix Tetrastigma hemsleyani flavone could inhibit the growth of CRC through the Wnt/β‐catenin signalling pathway [14]. Therefore, TCM may show excellent therapeutic potential in the treatment of CRC. Modern Chinese medicine suggests that CRC is initiated due to the long‐term accumulation of evil qi, such as ‘dampness heat’, ‘blood stasis’ and ‘retention of toxin stagnation’ in the intestine [15]. Tianma granule prescription is composed of Scolopendra, Scorpio, Herba Lobeliae chinensis, Phellodendron, Sparganium stolonierum, Buch.‐Ham., Arisaema cum bile, Seaweed, Astragalus membranaceus, Dioscoreae Rhizoma and Rhei Radix Et Rhizoma. It has the effects of attacking poison and expelling evil, resolving blood stasis and lumps, resolving phlegm and dissipating knots and dredging collaterals and relieving pain, with the primary effect of attacking poison and expelling evil, supplemented with body strengthening effects. Moreover, some active ingredients in Tianma granules have been shown to exhibit anti‐inflammatory and anti‐CRC effects. For example, Astragalus extract can inhibit CRC cell metastasis through the β‐Catenin‐mediated CXCR4 and EMT signalling pathway [16]. Astragalus polysaccharide can prevent the progression of experimental colitis [17]. Sulfated polysaccharides in seaweed could inhibit CRC cell vitality [18]. Therefore, Tianma granules may exert potential therapeutic effects on CRC.

This study explored the therapeutic effect of Tianma granules on tumours and its potential mechanism in the azoxymethane (AOM)/dextran sodium sulfate (DSS)‐induced CRC mouse model. Moreover, the inhibition of Tianma granules in the proliferation, migration and invasion of CRC cells was verified in vitro.

## 2 Materials and Methods

### 2.1 Preparation of Tianma Granules

Tianma granules are composed of Scolopendra (dried Scolopendridae), Scorpio (dried *Buthus martensii* Karsch), Herba Lobeliae chinensis (dried whole *Lobelia chinensis* Lour.), Phellodendri amurensis cortex (dried bark of *Phellodendron chinense* Schneid.), Sparganii Rhizoma Praeparata (dried tuber of *Sparganium stolonierum*, Buch.‐Ham. processed with vinegar), Arisaema cum bile ( *Arisaema heterophyllum* Blume fine powder mixed with beef, sheep, or pig's bile), seaweed [*Sargassum palidum* (Turn.) C. Ag dried algae], Astragalus membranaceus [dried rhizome of *Astragalus membranaceus* (Fisch.) Bge.], Dioscorea Rhizoma (dried tuber of *Dioscorea polystagya* Turczaninow) and Rhei Radix Et Rhizoma (dried rhizome of *Rheum palmatum* L.) (Table 1). All herbs were provided by the Pharmaceuticals Department of the First Affiliated Hospital of the Hunan University of Chinese Medicine. The aforementioned raw medicine (88 g in total) was placed in a 1000 mL round‐bottom flask, added with 10 times the amount of water, and soaked for 30 min, followed by 1 h of decoction. Next, after gauze filtering, eight times the amount of water was added for the second time, followed by 30 min of decoction. The two decoctions were mixed, rotary evaporated and concentrated. The granules were yielded by vacuum drying (88 g of raw medicine into 24.21 g of granules). The quality control of Tianma granules was performed by high‐performance liquid chromatography (HPLC)‐mass spectrometry (MS) (Figure S1). Briefly, the 3.0 g dried Tianma granules were resolved in 30 mL methanol with ultrasound. The supernatant was subsequently filtered with a 0.22 μm filter. HPLC was performed by the Agilent 1290UPLC system (Agilent, USA) with Agilent C18 (100 mm × 3.0 mm, 1.8 μm) at a flow rate of 0.4 mL/min. Mobile phase: acetonitrile (A) and aqueous solution (B). MS was performed on Agilent 6120 Single Quadrupole LC/MS System (Agilent) with both positive and negative ionisation modes with a capillary voltage of 4000 V, gas temperature of 350°C and 6.8 L/min flow rate. The ingredients of phellodendrine (reference standard for Phellodendri amurensis cortex), Astragaloside A (for Astragalus membranaceus), rheic acid and chrysophanol (for Rhei Radix Et Rhizoma) (Chengdu Pufei De Biotech Co. Ltd.) were set as reference standards.

**TABLE 1:** The composition of Tianma granules.

Herb or animal name	Chinese name	Bontany or zoology name	Application	Raw medicine weight (g)
Scolopendra	Wugong	Scolopendridae	Dried whole body	2
Scorpio	Quanxie	*Buthus martensii* Karsch	Dried whole body	3
Herba Lobeliae chinensis	Banbianlian	*Lobelia chinensis* Lour.	Dried whole herb	15
Sparganii Rhizoma Praeparata	Cusanleng	*Sparganium stolonierum*, Buch.‐Ham.	Dried tuber with vinegar processed	10
Rhei Radix Et Rhizoma	Dahuang	*Rheum palmatum* L.	Dried rhizome	6
Arisaema cum bile	Dannanxing	*Arisaema heterophyllum* Blume	Dried tuber powder mixed with beef, sheep or pig's bile for fermentation	6
Seaweed	Haizao	*Sargassum palidum* (Turn.) C. Ag	Dried rhizome	10
Phellodendri amurensis cortex	Huangbai	*Phellodendron chinense* Schneid.	Dried bark	6
Astragalus membranaceus	Huangqi	*Astragalus membranaceus* (Fisch.) Bge.	Dried tuber	20
Dioscorea Rhizoma	Shanyao	*Dioscorea polystagya* Turczaninow	Dried tuber	10

### 2.2 Establishment of Animal Model

**FIGURE 1:**
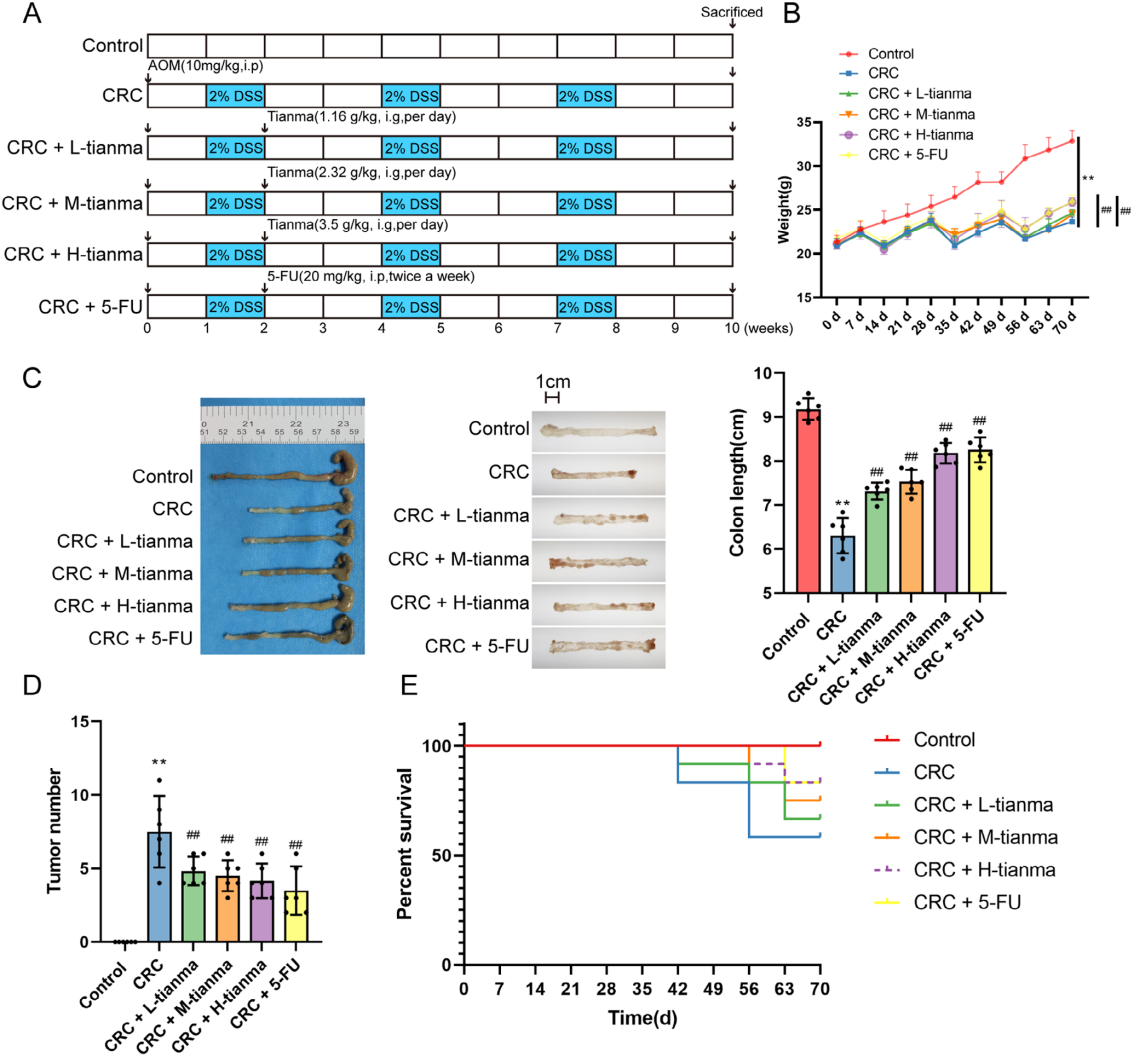
Tianma granules can improve the occurrence and development of CRC in AOM/DSS‐induced mice. (A) Schematic diagram of AOM/DSS model experimental program; (B) changes in mouse body weight were analysed; (C) colon length and (D) CRC tumour numbers in mice were analysed; (E) mouse survival rate was detected. *N* = 6. Data are expressed as mean ± SD. Statistical significance was determined by one‐way ANOVA followed by Tukey's multiple comparisons test for (B–D). Survival rate in (E) was analysed by Kaplan–Meier survival curves and log‐rank test. **p < 0.01, compared with the control group; ^##^ *p* < 0.01, compared with the CRC group.

All animal experiments were approved by the Animal Experiment Ethics Committee of Hunan University of Chinese Medicine. C57BL/6 mice (aged 6 weeks) were procured from the Hunan SJA Laboratory Animal Co. Ltd. (Changsha, Hunan, China). All the mice were housed under SPF conditions with a temperature of 22°C ± 1°C 55% ± 10% humidity and a 12 h/12 h light cycle. Mice were free to access water and food. After adaptive feeding for 1 week, the mice were randomly allocated into five groups: control (*n* = 10), CRC (*n* = 12), CRC + L‐tianma (*n* = 12), CRC + M‐tianma (*n* = 12), CRC + H‐tianma (*n* = 12) and CRC + 5‐Fu (*n* = 12) groups. On the first day of the experiment, all mice (except for mice in the control group) were intraperitoneally injected with one dose of 10 mg/kg azoxymethane (AOM). After injection for 1 week, 2% dextran sulfate sodium salt (DSS) was added to the drinking water of the mice for 7 days. Next, normal drinking water was resumed during the recovery period (14 days) for a total of three cycles. Starting from the first recovery period, mice in the CRC + L‐tianma, M‐tianma or H‐tianma groups were orally administered with low‐dose (1.16 g/kg), medium‐dose (2.32 g/kg) and high‐dose (3.5 g/kg) Tianma granules aqueous solution, respectively, every day. The high dose of mice was converted from clinical human equivalent doses [19] based on body surface area (9.1:1). Mice in the CRC + 5‐Fu group were intraperitoneally injected with 20 mg/kg 5‐Fu twice a week. Mice in the control group were injected with an equal dose of phosphate‐buffered saline (PBS) on the first day and drank normal drinking water daily. Mice were weighed once weekly. After 10 weeks, all mice were euthanised and their colon tissues were harvested. The schematic diagram of the experimental design is depicted in Figure 1A.

### 2.3 Histopathological Examination

The extracted intestine was rinsed with cold PBS and then measured for length. The intestine was then longitudinally incised for preliminary visual examination. Following the previously reported method [20], half of the intestines were fixed overnight in the Swiss‐rolling method in 4% paraformaldehyde. The samples were subsequently embedded in paraffin and then sectioned (4 μm). After gradient ethanol hydration, the sections were stained with haematoxylin and eosin (H&E; Wuhan Servicebio Co. Ltd., Wuhan, Hubei, China) following the instructions. Afterward, the sections were dehydrated using gradient ethanol, sealed with neutral gum, and then observed under an optical microscope (Nikon Corporation, Tokyo, Japan).

### 2.4 Alcian Blue‐Periodic Acid Schiff (AB‐PAS) Staining

The fixed colon was stained with AB‐PAS for analysis of goblet cells as per the kit's instructions (Nanjing JianCheng Bioengineering Institute, Nanjing, Jiangsu, China). In short, after hydration, the sections were stained with Alcian blue and periodic acid for 10 min, washed in distilled water, and then incubated with Schiff reagent for 5 min. The sections were subsequently stained with haematoxylin for 30 s. The sections were then sealed and observed under an optical microscope (Olympus, Japan).

### 2.5 Terminal Deoxynucleotidyl Transferase dUTP Nick‐End Labeling (TUNEL) Assay

CRC tissue cell apoptosis was detected using the TUNEL kit (Servicebio) as directed by the manufacturer. In short, after paraffin removal, the sections were incubated with protease K (37°C, 22 min) for antigen retrieval, permeabilised (room temperature, 20 min) with 0.1% Triton, and then incubated (10 min) with buffer. Next, the sections were added with TDT enzyme, dUTP, and buffer in a ratio of 1:5:50 for incubation (37°C, 1 h). The nucleus was subsequently counter‐stained (room temperature, 10 min) by DAPI in light‐deprived conditions. The sections were sealed with an anti‐fluorescence quenching sealing agent (Servicebio) and then observed under a fluorescence microscope (Olympus).

### 2.6 Enzyme‐Linked Immunosorbent Assay (ELISA)

TNF‐α (CSB‐E04744m), IL‐1β (CSB‐E08054m) and IL‐6 (CSB‐E04639m) ELISA kits were purchased from Wuhan CUSABIO Biotech Co. Ltd. (Wuhan, Hubei, China); INF‐γ (PI507) ELISA kit was supplied by Shanghai Beyotime Biotechnology Co. Ltd. (Shanghai, China). The colon tissues were homogenised and stored overnight at −20°C as directed by the manufacturer. The cell membrane ruptured after two freeze–thaw cycles. The homogenate was centrifuged (5000 *g*, 2°C–8°C, 5 min). The supernatant was used to detect the concentrations of TNF‐α, IL‐1β, IL‐6 and IFN‐γ using the corresponding ELISA kits. Finally, the optical density (OD) value at 450 nm was measured using an enzyme‐linked microreader (Bio‐rad, USA).

### 2.7 Oral Toxicity Study

For the oral toxicity study of Tianma granules, six C57BL/6 mice (6‐week‐old, male) were orally administered high‐dose Tianma granules once per day for 8 weeks. Then, the mice were euthanised and blood samples, heart, liver, spleen, lung and kidney samples were collected. After the blood clotted, the serum was isolated from the blood by centrifugation, and the levels of aspartate aminotransferase (AST), alanine aminotransferase (ALT), blood urea nitrogen (BUN) and creatinine (Cr) in the serum were determined by commercial kits (CSB‐E16539m, CSB‐E12649m; CUSABIO, Wuhan, China; C013‐3‐1, C011‐2‐1; Nanjing Jiancheng Bioengineering Institute). The organs were fixed in 4% paraformaldehyde and subjected to H&E staining as mentioned above.

### 2.8 Cell Culture and Treatment

Human CRC HCT116 (CCL‐247) and SW480 (CCL‐228) cells were purchased from the American Type Culture Collection (ATCC, Manassas, Virginia, USA). HCT116 and SW480 cells were cultured in RPMI‐1640 medium with 10% (v/v) fetal bovine serum (FBS) and 1% penicillin–streptomycin in a humidified incubator (37°C, 5% CO_2_). The cells were then treated with 0.2, 0.5 or 1 mg/mL Tianma granules [21] or berberine (12.5, 25 and 50 μg/mL [22]; Yuanye Biotech, Shanghai, China) (fully diluted with complete culture medium) for 48 h for 3‐(4,5‐dimethylthiazol‐2‐yl)‐2,5‐diphenyltetrazolium bromide (MTT) assay, and treated with 0.2, 0.5 or 1 mg/mL Tianma granules or 12.5, 25 and 50 μg/mL berberine for 24 h for other experiments.

### 2.9 MTT Assay

CRC cell viability was detected using an MTT assay. HCT116 and SW480 cells were seeded (3 × 10^3^ cells/well) onto 96‐well plates. After the cells underwent treatment with different concentrations of Tianma granules (0.2, 0.5 and 1 mg/mL) or berberine (12.5, 25 and 50 μg/mL) for 48 h, MTT solution (5 mg/mL) was added to the culture medium for incubation (37°C, 2 h). The culture medium was removed, and cells in each well were added to 100 μL DMSO. The OD value at 490 nm of cells in each well was measured using a microplate reader.

### 2.10 Colony Formation Assay

After treatment with different concentrations of Tianma granules or berberine, HCT116 and SW480 cells were seeded (2 × 10^3^ cells/well) into 6‐well plates and cultured for 2 weeks. After PBS washing, cells were fixed in 4% paraformaldehyde and stained with 5% crystal violet (Beyotime). The colonies were counted and photographed under an optical microscope (Olympus).

### 2.11 Flow Cytometry

Cell apoptosis was detected using the Annexin V‐FITC Cell Apoptosis Detection Kit (Beyotime) as per the instructions. In short, cells were centrifuged (1000 *g*, 5 min) with the supernatant discarded after 24‐h treatment with different concentrations of Tianma granules or berberine. After resuspension in PBS, 10^5^ resuspended cells were centrifuged (1000 *g*, 5 min), with the supernatant discarded, followed by the addition of 195 μ L buffer for cell resuspension. Five micolitres Annexin V‐FITC and 10 μL PI staining were subsequently added for incubation (room temperature, 20 min) in light‐deprived conditions. Cell cycle was detected using PI staining. Briefly, cells were collected and fixed with 70% ethanol overnight at 4°C. The cells were then further stained with PI + RNase A solution in light‐deprived conditions for 30 min at 37°C after washing with cold PBS. Cell apoptosis and cell cycle distribution were detected using flow cytometry (Novocyte, Agilent, USA) within 1 h.

### 2.12 Scratch Assay

HCT116 and SW480 cells were seeded onto 6‐well plates (5 × 10^5^ cells/well), and the cells were confluent to 100%. The cells were treated with different concentrations of Tianma granules or berberine, and photographed after scratching for 24 and 48 h. The migration distance and migration rate were analysed using the Image J software.

### 2.13 Transwell Assay

HCT116 and SW480 cells were resuspended in 0.1% BSA‐containing serum‐free medium and the cell density was adjusted to 2 × 10^5^ cells/mL. The matrix‐coated upper chamber was added with the culture medium containing the cells; the lower chamber was filled with 600 μL RPMI‐1640 (with 10% FBS). After 24 h of treatment with different concentrations of Tianma granules or berberine, the cells on the lower side of the insert membrane were fixed and stained with crystal violet solution for 20 min. The invaded cells were observed under a microscope.

### 2.14 Senescence‐Associated β‐Galactosidase (SA‐β‐Gal) Staining

The cell senescence was performed by the In Situ β‐Gal Staining kit (Beyotime). HCT116 and SW480 cells were treated with different concentrations of Tianma granules and then fixed with the fixative solution for 10 min at room temperature. After being washed with PBS, cells were further incubated with a β‐Gal staining working solution for 2 h at 37°C. Finally, cells were captured using an optical microscope, and the number of positive cells was counted using ImageJ software (NIH, MD, USA).

### 2.15 Western Blot

The total protein of mouse colon tissues or CRC cells (HCT‐116 and SW480) was extracted using RIPA lysate (Beyotime). The BCA protein concentration detection kit (Beyotime) was used to determine protein concentration. The proteins were separated by sodium dodecyl sulfate‐polyacrylamide gel electrophoresis (SDS‐PAGE) and then transferred to polyvinylidene fluoride (PVDF) membranes (Millipore, USA). The membranes were blocked in 5% skim milk in a TBST solution for 1 h. The membranes were incubated (overnight, 4°C) with anti‐COX2 (1:1000, ab179800; Abcam Inc., Cambridge, MA, USA), anti‐MUC2 (1:1000, ab272692; Abcam), anti‐Bax (1:1000, ab32503; Abcam), anti‐Cleaved caspase3 (1:1000, #9661; Cell Signalling Technology, Beverly, MA, USA), anti‐AXIN1 (1:1000, #2087; Cell Signalling Technology), anti‐β‐catenin (1:5000, ab32572; Abcam), anti‐Wnt5a (1:1000, MA5‐14946; Invitrogen Inc., Carlsbad, CA, USA), anti‐Cyclin D1 (1:200, ab16663; Abcam), p‐GSK‐3β (67558‐1‐Ig; Proteintech), c‐Myc (AF6054; Affinity Bioscience, Changzhou, China) Lamin B1 (12987‐1‐AP; Proteintech), CDK4 (11026‐1‐AP; Proteintech), CDK6 (14052‐1‐AP; Proteintech) and anti‐GAPDH (1:2500, ab9485; Abcam). Following three washings with TBST solution, the membranes were incubated (room temperature, 2 h) with horseradish peroxidase (HRP)‐conjugated goat anti‐rabbit IgG antibody (1:2000, ab205718; Abcam). GAPDH served as an internal reference. The protein bands were visualised using the ECL chemiluminescence reagent kit (New Cell & Molecular Biotech Co. Ltd., Suzhou, China) and then quantified using Image J (NIH, Bethesda, Maryland, USA).

### 2.16 Network Pharmacology Analysis

GSE10950 contained expression profiles of 14 human CRC tissues and paired normal colon tissues. The differential gene expression analysis was performed using the GEO2R package to identify genes significantly expressed differently between CRC and normal samples. Genes with an absolute log_2_ fold change (∣logFC∣) > 1 and a *p*‐value less than 0.05 were considered differentially expressed (DEG).

The main active components of the following herbs composing Tianma granules were retrieved from the Integrated Traditional Chinese Medicine (ITCM) platform (http://itcm.biotcm.net). *Scolopendra*, *Scorpio*, *Herba Lobeliae chinensis*, *Phellodendri amurensis cortex*, *Sparganii Rhizoma Praeparata*, *Arisaema cum bile*, *Sargassum palidum*, *Astragalus membranaceus*, *Dioscorea Rhizoma* and *Rhei Radix Et Rhizoma*. Their corresponding predicted target genes were also obtained from this database.

The PubChem database (https://pubchem.ncbi.nlm.nih.gov/) was mined to expand the list of potential drug‐target genes. Concurrently, colorectal cancer‐related genes were retrieved from the SymMap database (http://www.symmap.org/) using the keyword ‘Colorectal cancer’. Then, the colorectal cancer‐related genes interacted with drug target genes and DEG from GSE10950. The interacted genes were subjected to Kyoto Encyclopedia of Genes and Genomes (KEGG) and Gene Ontology (GO) enrichment analysis using the KOBAS online tool (http://kobas.cbi.pku.edu.cn/). A protein–protein interaction (PPI) network of the interacted target genes was constructed using the STRING database (https://www.string‐db.org/). Interactions with a combined confidence score greater than 0.7 were included. The hub genes within the constructed network, characterised by a high degree of connectivity, were identified using the CytoHubba plugin (Degree algorithm) in Cytoscape software (version 3.9.1).

A Sankey diagram was generated to visually represent the complex relationships between the key active components of Tianma granules, their identified hub target genes, and the relevant signalling pathways involved in the therapeutic effect against colorectal cancer. The width of the connecting lines in the diagram was used to illustrate the relative strength or importance of the associations, with wider lines indicating a stronger connection.

### 2.17 Statistical Analysis

The yielded experimental data were expressed as mean ± SD. One‐way analysis of variance (ANOVA) and Student's *t*‐test were performed using GraphPad Prism 8.0 software. The Tukey method was used for multiple comparisons. A *p*‐value of less than 0.05 indicated a statistically significant difference.

## 3 Results

### 3.1 Tianma Granules Can Improve CRC Occurrence and Development in AOM/DSS‐Induced Mice

Firstly, the AOM/DSS‐induced CRC mouse model was established, and the anti‐tumour effect of Tianma Granules on CRC mice was determined; 5‐Fu was used as the positive treatment control (Figure 1A). According to the results, AOM/DSS‐induced CRC mice showed significant weight loss (Figure 1B) and colon shortening (Figure 1C) compared to the control mice. Moreover, polypoid tumours were found in the middle and end of the colon in CRC mice (Figure 1D), accompanied by a decreased survival rate (Figure 1E).

Compared to the CRC group, after treatment with low‐dose (L‐tianma), medium‐dose (M‐tianma), or high‐dose (H‐tianma) Tianma granules, or 5‐Fu, the mice showed restored body weight (Figure 1B). Furthermore, treatment with L‐tianma, M‐tianma, H‐tianma, or 5‐Fu significantly reduced colon shortening (Figure 1C) and decreased tumour numbers (Figure 1D) compared to the CRC group. The survival rate was also improved in mice treated with L‐tianma, M‐tianma, H‐tianma, or 5‐Fu compared to the CRC group (Figure 1E). Among the Tianma granule groups, H‐tianma demonstrated the most significant improvements in colon length and tumour numbers. It can be reasonably concluded from the above findings that tianma granules can alleviate the occurrence and development of CRC tumours in AOM/DSS‐induced mice. Moreover, oral toxicity analysis showed that 8 weeks of oral administration of H‐tianma did not induce significant alteration in body weight, histopathology of major organs (heart, liver, spleen, lung and kidney) and hepatic/renal function (AST, ALT, Cr, BUN, *p* > 0.05 vs. control) (Figure S2).

### 3.2 Tianma Granules Alleviated the Pathological Damage and Inflammation of CRC Mice and Promoted CRC Cell Apoptosis

**FIGURE 2:**
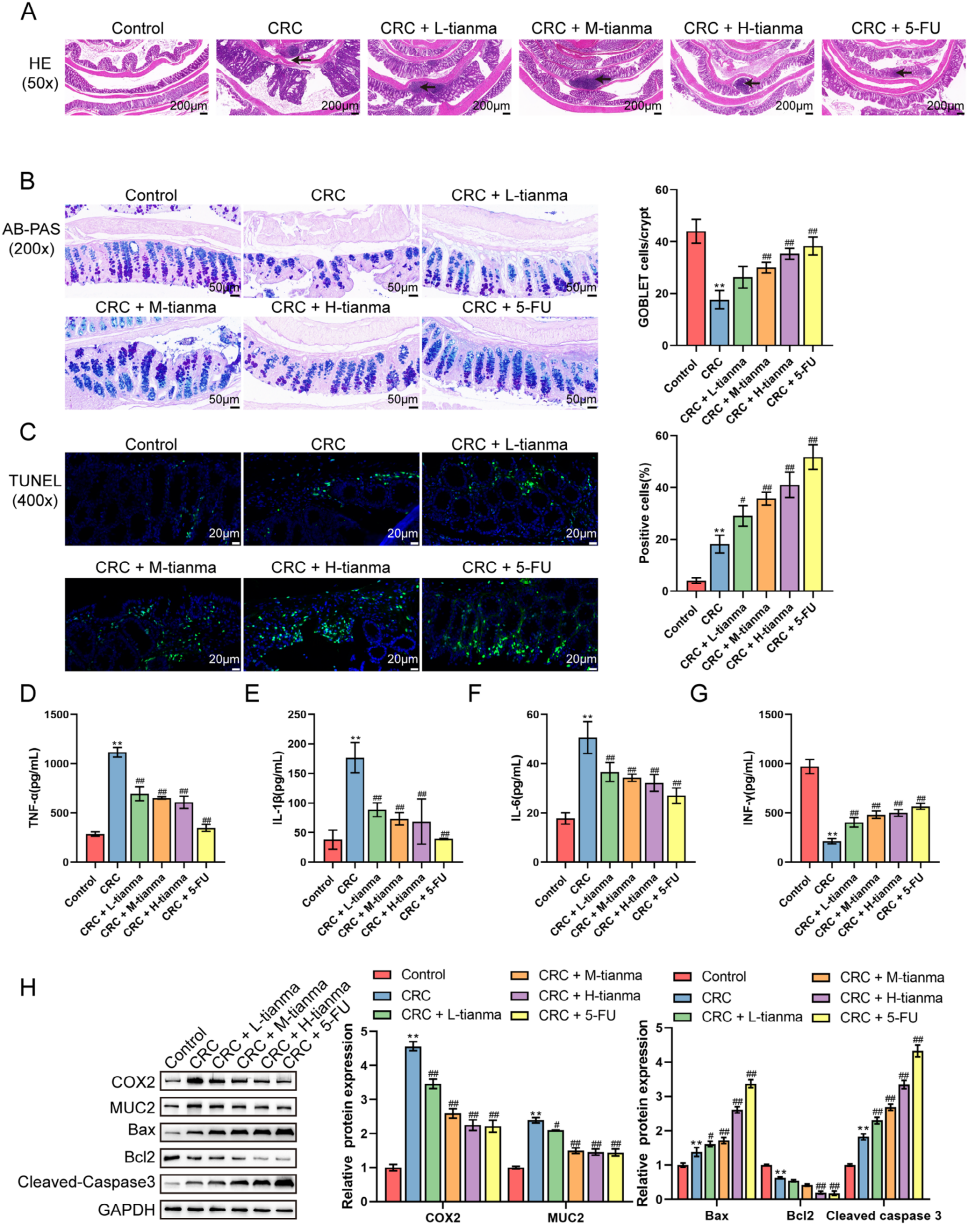
Tianma granules alleviated the pathological damage and inflammation of CRC mice and promoted CRC cell apoptosis. (A) H&E staining was used to detect the pathological damage of colon tissues (black arrows indicated tumours in the colon). (B) AB‐PAS staining was used to detect and quantify goblet cell numbers in colon tissues. (C) TUNEL staining was used to detect cell apoptosis and the apoptotic cell rate in colon tumour tissues (green fluorescence represented apoptotic cells). (D–G) ELISA was used to detect the levels of inflammatory cytokines (TNF‐α, IL‐1β, IL‐6 and IFN‐γ) in colon tissues. (H) Western blot was used to detect the protein levels of CRC‐specific proteins (COX2 and MUC2) and apoptotic proteins (Bax, Cleaved‐caspase3 and Bcl2). *N* = 3. Data are expressed as mean ± SD. Statistical significance was determined by one‐way ANOVA followed by Tukey's multiple comparisons test. **p < 0.01, compared with the control group; ^#^ *p* < 0.05, ^##^ *p* < 0.01, compared with the CRC group.

Furthermore, the effects of Tianma granules on colorectal inflammation and histopathology in CRC mice were detected. As indicated by H&E staining results, the colonic tissue of AOM/DSS‐induced CRC mice demonstrated inflammatory cell infiltration, accompanied by loss of crypt structure and goblet cells, and reduced intestinal epithelial cells in the colon (Figure 2A). AB‐PAS staining results showed that CRC mice exhibited significantly reduced goblet cell numbers (Figure 2B). TUNEL staining was used to detect the apoptosis rate of cells in the mouse colon. The results revealed that the apoptosis rate of cells in CRC mice was significantly increased (Figure 2C). Furthermore, the inflammatory factors in colorectal tissues were detected using ELISA; TNF‐α, IL‐1β and IL‐6 levels were elevated, and IFN‐γ level was decreased (Figure 2D–G). Western blot was used to detect the levels of CRC‐specific proteins (COX2 and MUC2) and apoptotic proteins (Bax, Cleaved‐caspase3 and Bcl2); it was found that CRC mice showed increased protein levels of COX2, MUC2, Bax and Cleaved caspase3, and decreased Bcl2 level (Figure 2H). Compared to the CRC group, L‐tianma, M‐tianma, or H‐tianma granules, or 5‐Fu treatments could effectively reduce inflammatory cell infiltration and tumour cell numbers while increasing goblet cell numbers in the colon (Figure 2A,B). After treatment with L‐tianma, M‐tianma, H‐tianma, or 5‐Fu, compared to the CRC group, the apoptosis rate of CRC tissue cells was increased (Figure 2C). Moreover, compared to the CRC group, L‐tianma, M‐tianma, H‐tianma, or 5‐Fu treatments reduced TNF‐α, IL‐1β and IL‐6 levels, and increased IFN‐γ levels (Figure 2D–G). Additionally, compared to the CRC group, COX2, MUC2 and Bcl2 protein levels were decreased, while Bax and Cleaved caspase3 levels were increased after treatment with L‐tianma, M‐tianma, H‐tianma, or 5‐Fu (Figure 2H). Among the Tianma granule groups, H‐tianma consistently showed the most significant improvements in these inflammatory and apoptotic markers. From all the above, Tianma granules can alleviate pathological damage and inflammation of CRC mice and promote CRC cell apoptosis in a dose‐dependent manner.

### 3.3 Tianma Granules Can Inhibit CRC Cell Proliferation and Promote Cell Apoptosis In Vitro

**FIGURE 3:**
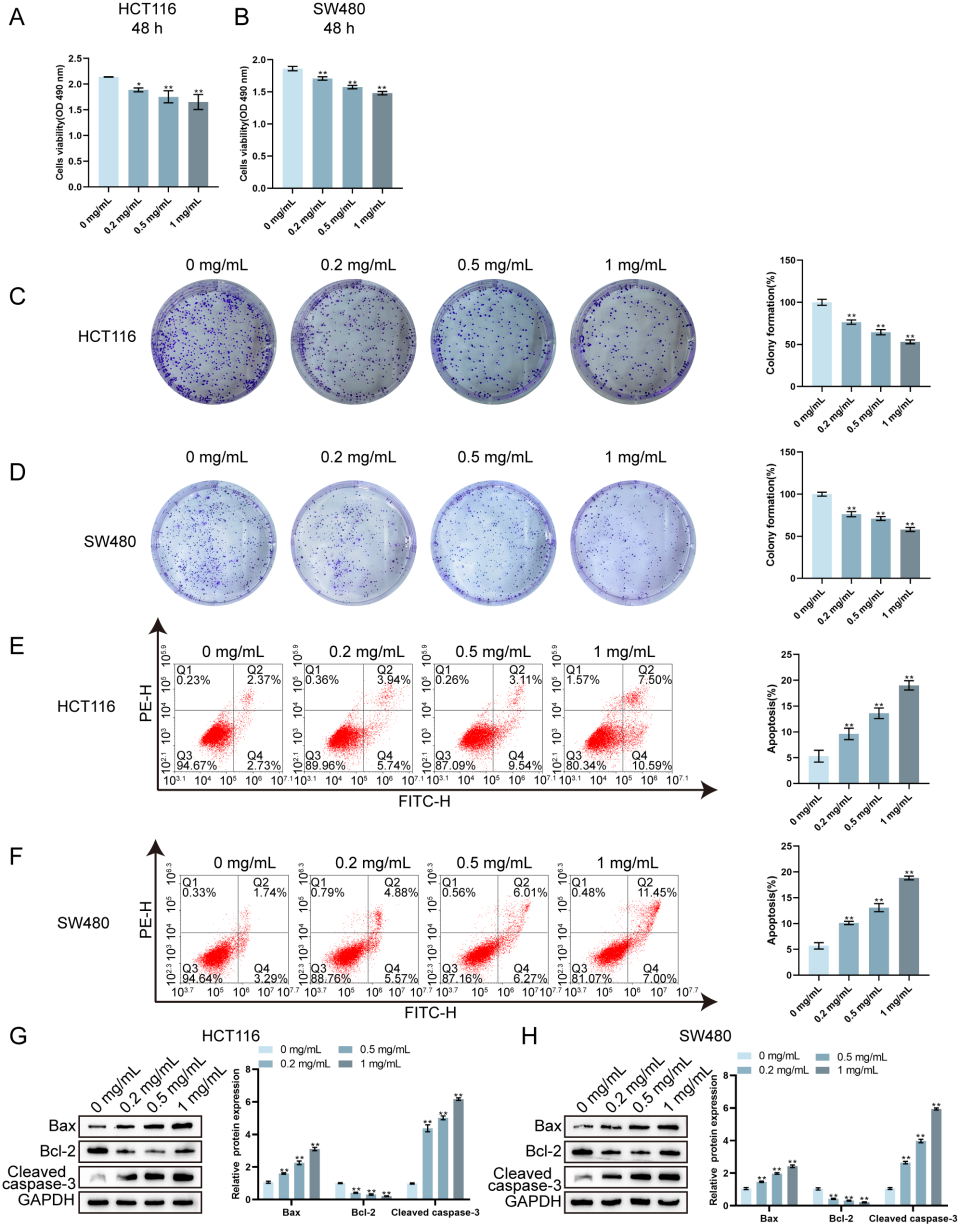
Tianma granules can inhibit CRC cell proliferation and promote cell apoptosis in vitro. After treating HCT116 and SW480 cells with different concentrations of Tianma granules (0.2, 0.5 and 1 mg/mL) for 48 h, MTT assay was used to detect the cell viability of HCT116 cells (A, B) and SW480 cells; (C, D) colony formation assay was used to detect CRC cell proliferation; (E, F) flow cytometry was used to detect CRC cell apoptosis; (G, H) western blot was used to detect the protein levels of Bax, cleaved caspase3 and Bcl‐2 in CRC cells. *N* = 3. Data are expressed as mean ± SD. Statistical significance was determined by one‐way ANOVA followed by Tukey's multiple comparisons test. *p < 0.05, **p < 0.01.

Subsequently, the effects of Tianma granules on the proliferation and apoptosis of CRC cells were verified in vitro. Firstly, HCT116 and SW480 cells were treated with different concentrations of Tianma granules (0.2, 0.5 and 1 mg/mL). According to MTT assay and plate colony formation assay results, Tianma granules noticeably inhibited HCT116 and SW480 cell viability and proliferation (Figure 3A–D). Moreover, Tianma granules remarkably promoted HCT116 and SW480 cell apoptosis as indicated by flow cytometry results (Figure 3E,F). Western blot was used to analyse the apoptosis‐related proteins (Bax, Cleaved‐caspase3 and Bcl2). It was observed that Tianma granules notably promoted Bax and Cleaved caspase3 protein levels while inhibiting Bcl‐2 levels in HCT116 and SW480 cells (Figure 3G,H). These results indicate that Tianma granules can inhibit the proliferation of CRC cells and promote cell apoptosis in vitro.

### 3.4 Tianma Granules Inhibited CRC Cell Migration and Invasion In Vitro

**FIGURE 4:**
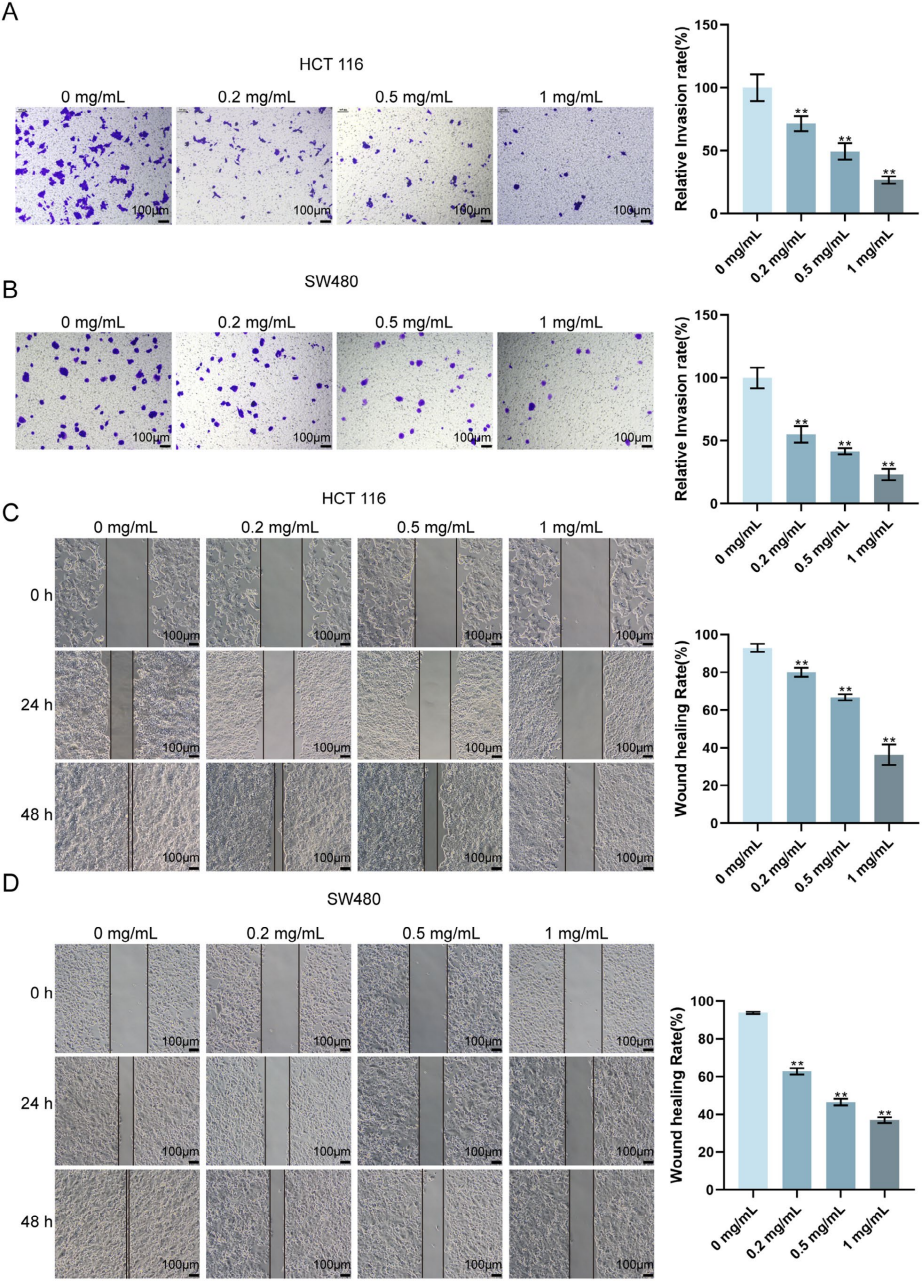
Tianma granules inhibited CRC cell migration and invasion in vitro. After treating HCT116 and SW480 cells with different concentrations of Tianma granules (0.2, 0.5 and 1 mg/mL) for 24 h, Transwell assay was used to detect the invasion of (A) HCT116 cells and (B) SW480 cells; scratch assay was used to detect the migration ability of (C) HCT116 cells and (D) SW480 cells. *N* = 3. Data are expressed as mean ± SD. Statistical significance was determined by one‐way ANOVA followed by Tukey's multiple comparisons test. **p < 0.01.

The effects of Tianma granules on the migration and invasion of CRC cells were subsequently explored in vitro. As shown by Transwell assay results, Tianma granules significantly inhibited the invasion of HCT116 and SW480 cells (Figure 4A,B). The scratch assay results revealed that Tianma granules reduced the migration distance of HCT116 and SW480 cells (Figure 4C,D). The results above indicate that Tianma granules can inhibit the migration and invasion of CRC cells.

### 3.5 Tianma Granules Promoted CRC Cell Senescence and Cell Cycle Arrest

**FIGURE 5:**
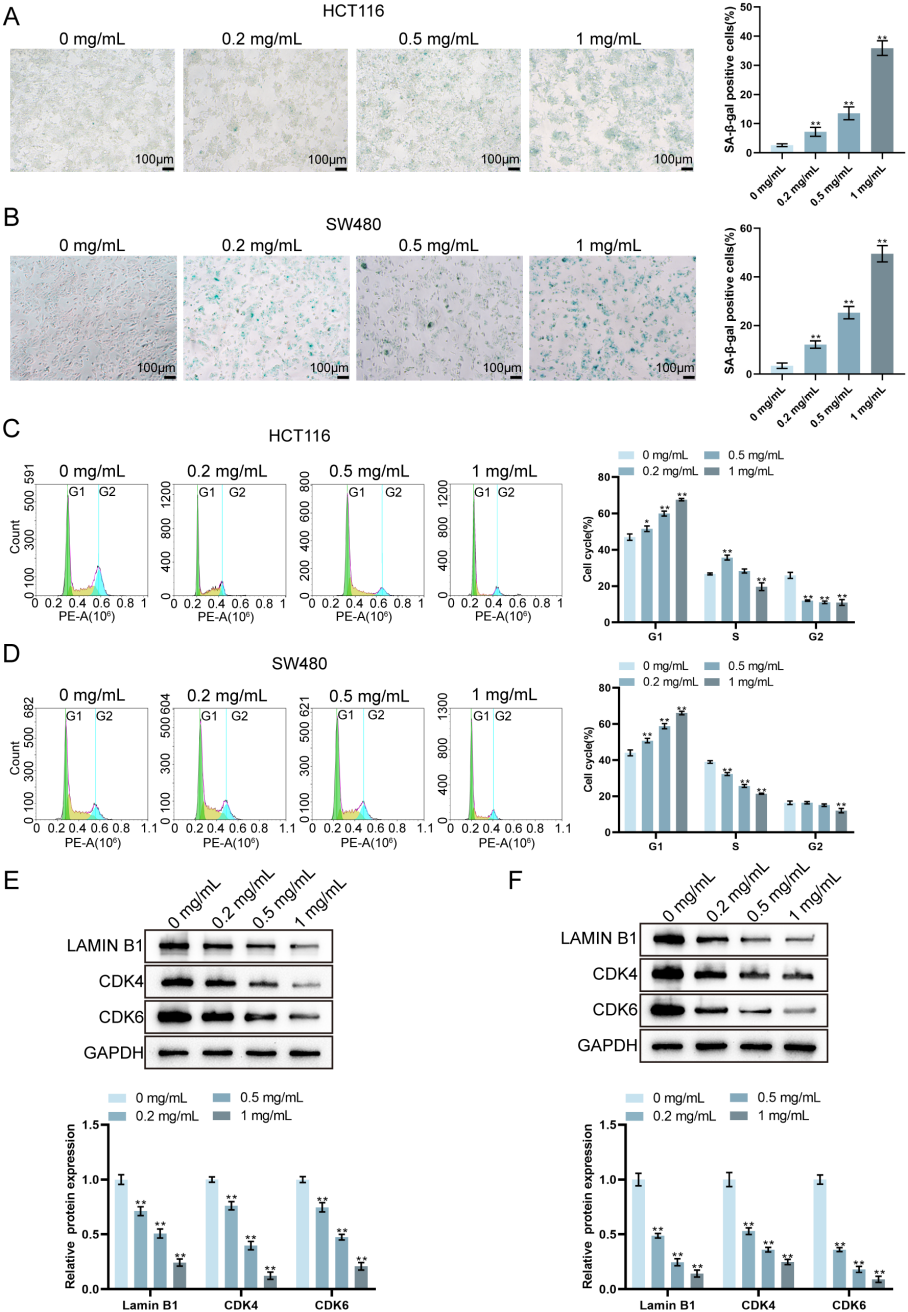
Tianma granules promoted CRC cell senescence and cell cycle arrest in vitro. (A, B) HCT116 (A) and SW480 (B) cells were treated with Tianma granules at concentrations of 0, 0.2, 0.5 and 1 mg/mL. Representative images are shown on the left and quantitative analysis of SA‐β‐gal‐positive cells is shown on the right. (C, D) Flow cytometry was used to analyse the cell cycle distribution in HCT116 (C) and SW480 (D) cells treated with different concentrations of Tianma granules. The percentages of cells in the G1, S and G2 phases are shown in the histograms on the right. (E, F) HCT116 (E) and SW480 (F) cells were treated with Tianma granules at the indicated concentrations and the protein levels of lamin B1, CDK4 and CDK6 were determined by western blot. *N* = 3. Data are expressed as mean ± SD. Statistical significance was determined by one‐way ANOVA followed by Tukey's multiple comparisons test. *p < 0.05, **p < 0.01.

Cell senescence and cell cycle distribution in HCT116 and SW480 cells treated with varying concentrations of Tianma granules (0, 0.2, 0.5 and 1 mg/mL) were examined to further explore the effect of Tianma granules on CRC cells. SA‐β‐gal staining revealed that treatment with Tianma granules resulted in a significant increase in SA‐β‐gal‐positive cells in both HCT116 and SW480 cell lines in a dose‐dependent manner, with the highest concentration of 1 mg/mL markedly elevating the number of senescent cells compared to the control group (0 mg/mL; Figure 5A,B). A flow cytometry analysis indicated that Tianma granules induced cell cycle arrest in both CRC cell lines, leading to an accumulation of cells in the G1 phase and a decrease in the S and G2 phases, with more significant effects at higher concentrations of Tianma granules and notable differences observed between treated and control groups (Figure 5C,D). Western blot analysis of cell cycle regulators Lamin B1, CDK4 and CDK6 showed a dose‐dependent decrease in the expression of all three factors in both HCT116 and SW480 cells after treatment with Tianma granules (Figure 5E,F). These results suggest that Tianma granules promote cellular senescence and induce cell cycle arrest in CRC cells, thereby inhibiting their proliferation.

### 3.6 Tianma Granules Inhibited the Activation of the Wnt Pathway in CRC Mice and Cells

**FIGURE 6:**
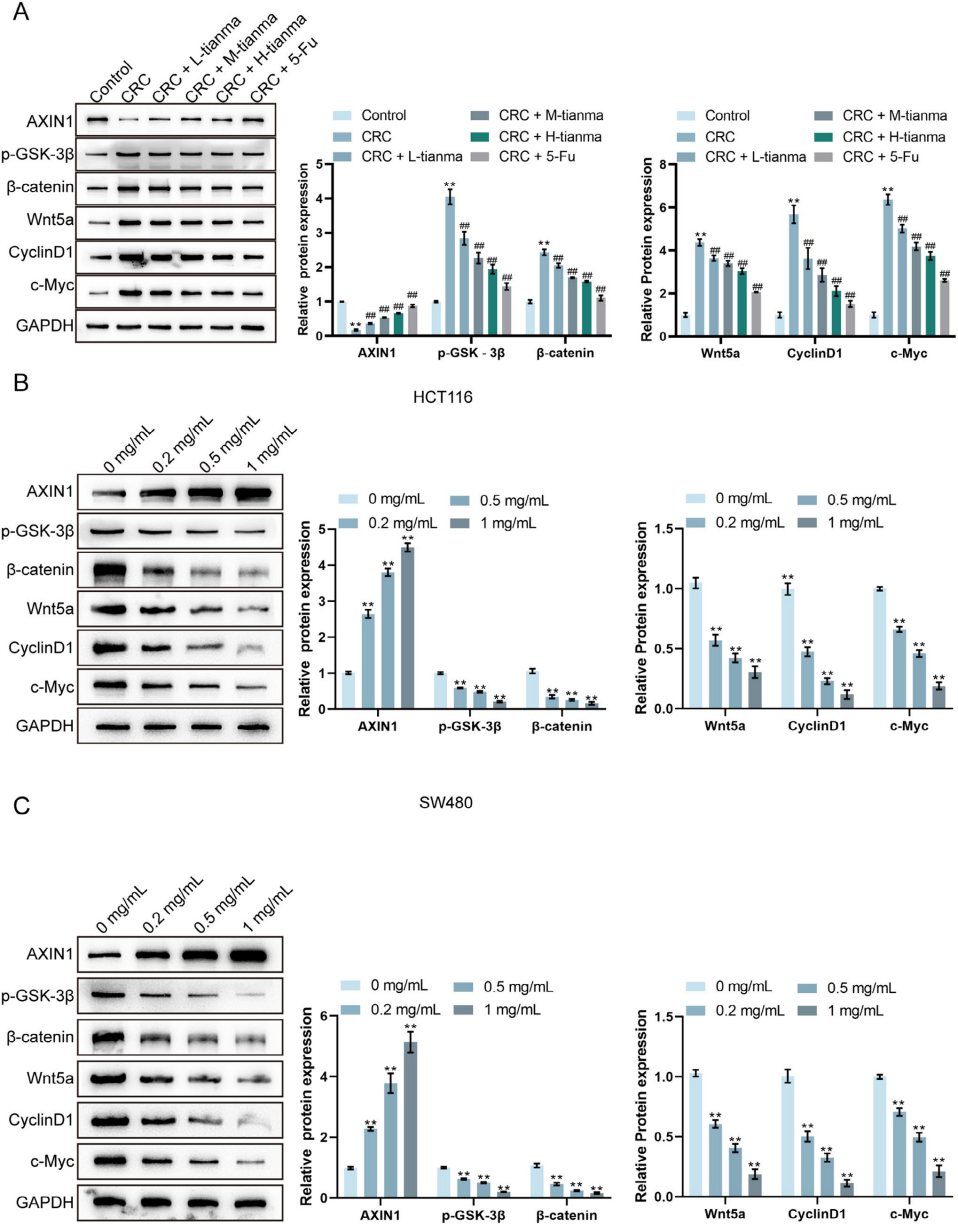
Tianma granules inhibited the activation of the Wnt pathway in CRC mice and cells. (A) Western blot analysis was used to detect the protein levels of AXIN1, p‐GSK‐3β, β‐catenin, Wnt5a, CyclinD1 and c‐Myc in the colon tissues of AOM/DSS‐induced CRC mice. (B) After treating HCT116 cells with different concentrations of Tianma granules (0.2, 0.5 and 1 mg/mL) for 24 h, western blot analysis was used to detect the protein levels of AXIN1, p‐GSK‐3β, β‐catenin, Wnt5a, CyclinD1 and c‐Myc. (C) Western blot analysis was performed to detect the protein levels of AXIN1, p‐GSK‐3β, β‐catenin, Wnt5a, CyclinD1 and c‐Myc in SW480 cells treated with different concentrations of Tianma granules (0.2, 0.5 and 1 mg/mL) for 24 h. GAPDH served as a loading control. *N* = 3. Data are expressed as mean ± SD. Statistical significance was determined by one‐way ANOVA followed by Tukey's multiple comparisons test. **p < 0.01, compared with the control/0 mg/mL group; ^##^ *p* < 0.01, compared with the CRC group.

The regulatory mechanism of Tianma granules in CRC was subsequently explored. A previous study concluded that the inhibition of the Wnt pathway is conducive to the prevention and treatment of CRC [23]. Therefore, the Wnt pathway‐related protein levels in the colon tissues of CRC mice were detected using Western blot in this study. It was observed that, compared to the control group, AXIN1 protein level was significantly reduced in CRC tissues, while p‐GSK‐3β, β‐catenin, Wnt5a, CyclinD1 and c‐Myc levels were significantly increased (Figure 6A). After treatment with L‐tianma, M‐tianma, or H‐tianma granules, or 5‐Fu, compared to the CRC group, these trends were reversed: AXIN1 levels were significantly increased, while p‐GSK‐3β, β‐catenin, Wnt5a, CyclinD1 and c‐Myc levels were significantly decreased (Figure 6A). The high‐dose Tianma granules generally induced the most profound changes among the Tianma treatment groups.

Similarly, Tianma granules also effectively modulated the Wnt/β‐catenin pathway in CRC cells (HCT116 and SW480 cells). Compared to the untreated control group (0 mg/mL Tianma granules), treatment with Tianma granules (0.2, 0.5 and 1 mg/mL) for 24 h significantly increased AXIN1 protein levels in both HCT116 (Figure 6B) and SW480 cells (Figure 6C) in a dose‐dependent manner. Concurrently, β‐catenin, Wnt5a, CyclinD1 and c‐Myc levels were significantly decreased in a dose‐dependent manner in both HCT116 (Figure 6B) and SW480 cells (Figure 6C) compared to the untreated control. The phosphorylation of GSK‐3β (p‐GSK‐3β) also showed a dose‐dependent decrease with Tianma granule treatment in both cell lines. The highest concentration of Tianma granules (1 mg/mL) induced the most significant changes in these protein expressions. Tianma granules can collectively inhibit the activation of the Wnt/β‐catenin pathway in CRC mice and cells in a dose‐dependent manner.

### 3.7 Network Pharmacology Analysis of Tianma Granules for Colorectal Cancer Treatment

**FIGURE 7:**
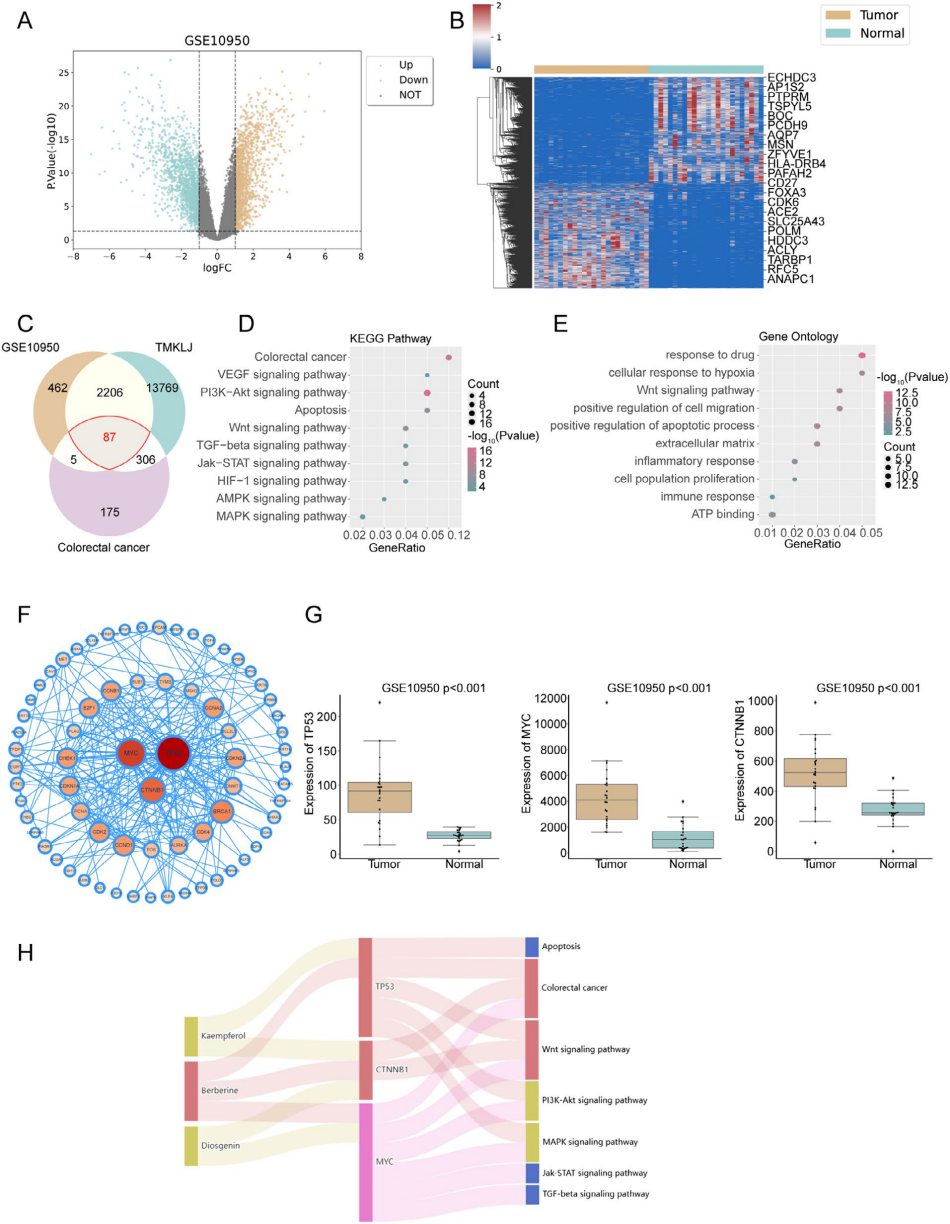
Network pharmacology analysis of Tianma granules for colorectal cancer treatment. (A) Volcano plot of differentially expressed genes in the GSE10950 dataset, showing upregulated (up) and downregulated (down) genes with ∣logFC∣ > 1 and *p* < 0.05. (B) Heatmap illustrating the expression profile of differentially expressed genes in GSE10950 colorectal cancer (tumor) and paired normal colon tissue (normal) samples. (C) Venn diagram showing the overlap among differentially expressed genes from GSE10950, predicted drug‐target genes from Tianma granules (TMKLJ), and colorectal cancer‐related genes. (D) KEGG pathway enrichment analysis of the common target genes. (E) Gene Ontology (GO) enrichment analysis of the common target genes. (F) Protein–protein interaction (PPI) network of the common target genes, with node size representing the degree of connectivity. (G) Expression levels of hub genes (TP53, MYC, CTNNB1) in colorectal cancer (tumour) and normal (normal) samples from the GSE10950 dataset. (H) Sankey diagram illustrating the relationship between key active components of Tianma granules, their hub targets, and relevant signalling pathways in colorectal cancer. The width of the connecting lines corresponds to the strength of the association.

A comprehensive network pharmacology analysis was conducted to elucidate the potential mechanisms of Tianma granules against colorectal cancer (CRC). First, a differential gene expression analysis using the GSE10950 dataset (24 CRC samples and 24 paired normal colon tissues) identified 2760 differentially expressed genes (∣logFC∣ > 1, *p* < 0.05) between CRC and normal samples (Figure 7A,B). Next, the main active components of various TCM herbs in Tianma granules (including Scolopendra, Scorpio, Herba Lobeliae chinensis, Phellodendron, Sparganium stolonierum, Arisaema cum bile, Seaweed, Astragalus membranaceus, Dioscoreae Rhizoma and Rhei Radix Et Rhizoma) were analysed through the ITCM platform. Subsequent PubChem mining yielded 16,386 predicted drug‐target genes. Concurrently, 573 CRC‐related genes were retrieved from the SymMap database based on the keyword ‘Colorectal cancer’. A Venn diagram identified 87 common genes among the differentially expressed genes, drug‐target genes and CRC‐related genes (Figure 7C). The KOBAS online analysis of these 87 common target genes revealed significant enrichment in both GO terms and KEGG pathways. KEGG pathway analysis indicated significant enrichment in pathways including the PI3K‐Akt signalling pathway, colorectal cancer, apoptosis, Wnt signalling pathway, Jak–STAT signalling pathway, MAPK signalling pathway, TGF‐β signalling pathway, HIF‐1 signalling pathway, VEGF signalling pathway and AMPK signalling pathway (Figure 7D). The GO analysis showed significant associations with biological processes such as response to drugs, positive regulation of cell migration, extracellular matrix, positive regulation of apoptotic process, response to hypoxia, ATP binding, inflammatory response, Wnt signalling pathway, cell population proliferation and immune response (Figure 7E). Notably, both GO and KEGG analyses consistently enriched the Wnt signalling pathway.

A protein–protein interaction (PPI) network was constructed for the 87 common target genes using the STRING database (Figure 7F). The top three hub genes identified were TP53, MYC and CTNNB1. Validation using the GSE10950 chip confirmed that TP53, MYC and CTNNB1 were significantly upregulated in colorectal cancer samples (Figure 7G). Furthermore, enrichment analysis indicated that TP53, MYC and CTNNB1 are all associated with the Wnt signalling pathway.

An investigation delving into the active components targeting these hub genes concluded that TP53 is targeted by Kaempferol and Berberine. MYC is targeted by Berberine and Diosgenin. CTNNB1 is targeted by Kaempferol, Berberine and Diosgenin. Among these, Berberine was identified as a key component, simultaneously targeting all three hub factors: TP53, MYC and CTNNB1. A Sankey diagram visually summarised these relationships, suggesting that the Berberine–MYC–Wnt pathway may represent a primary mechanism of action for Tianma granules, as indicated by the broadest connecting line (Figure 7H).

### 3.8 Berberine Inhibits CRC Cell Proliferation and Promotes Apoptosis In Vitro

**FIGURE 8:**
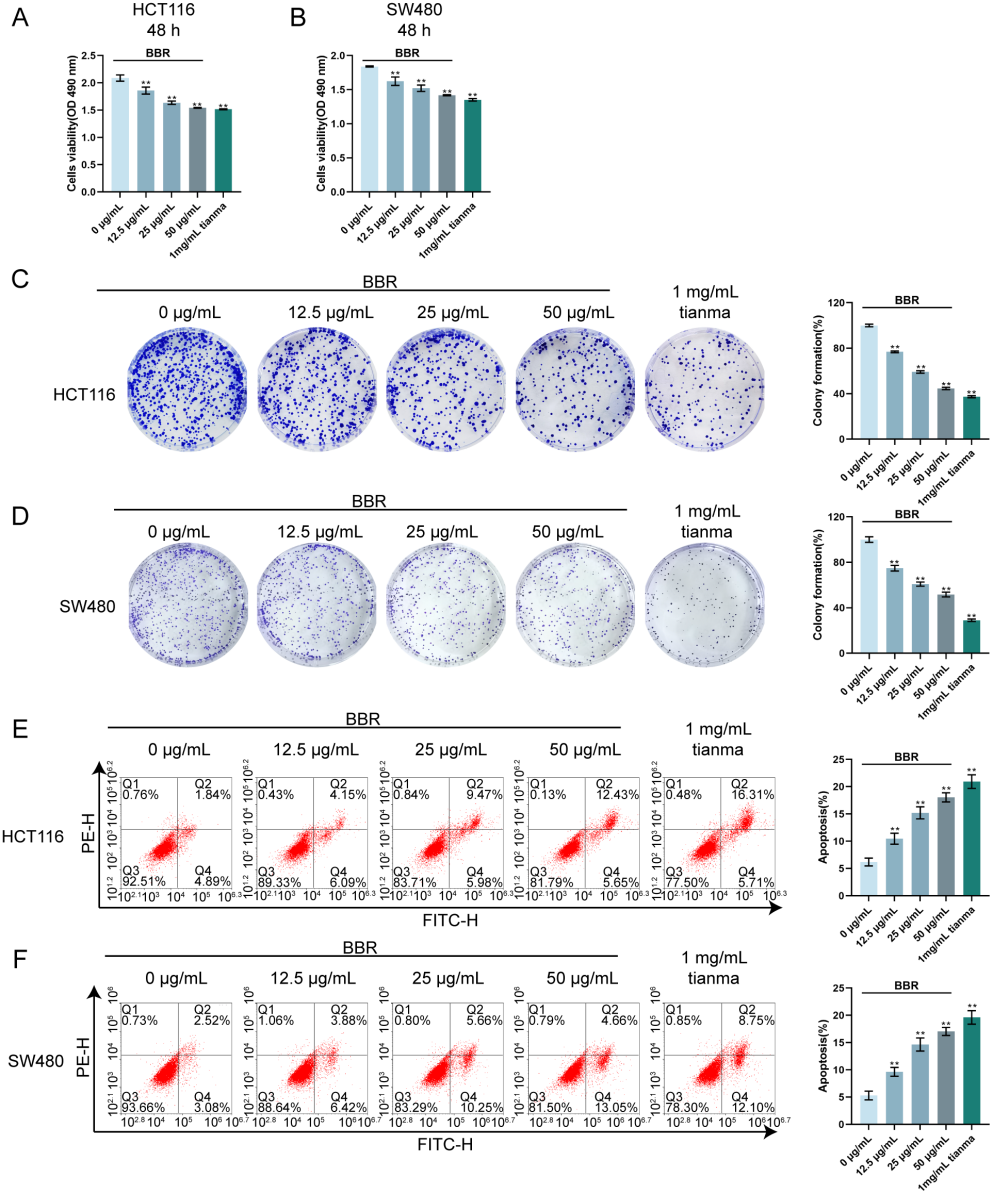
Berberine inhibits CRC cell proliferation and promotes apoptosis in vitro HCT116 and SW480 cells were treated with different concentrations of berberine (BBR: 12.5, 25 and 50 μg/mL) or 1 mg/mL Tianma granules. (A, B) Cell viability of HCT116 and SW480 cells after 48 h treatment, as determined by MTT assay. (C, D) Colony formation assay results for HCT116 and SW480 cells, showing representative images (left) and quantification of colony formation rate (right). (E) Flow cytometry analysis of apoptosis in HCT116 and SW480 cells, showing representative plots (left) and quantification of apoptotic percentage (right). *N* = 3. Data are expressed as mean ± SD. Statistical significance was determined by one‐way ANOVA followed by Tukey's multiple comparisons test. **p < 0.01, compared with the 0 μg/mL BBR group.

To further investigate the role of identified active components, the effects of berberine (BBR), a key compound identified in the network pharmacology analysis, on CRC cell proliferation and apoptosis were examined in vitro. HCT116 and SW480 cells were treated with varying concentrations of BBR (12.5, 25 and 50 μg/mL) or 1 mg/mL Tianma granules as a positive control. MTT assay results showed that both BBR and Tianma granules significantly inhibited the viability of HCT116 (Figure 8A) and SW480 cells (Figure 8B) after 48 h of treatment. Specifically, treatment with BBR caused a dose‐dependent decrease in cell viability for both HCT116 and SW480 cells compared to the control group (0 μg/mL BBR). The most significant inhibition of cell viability was observed with 1 mg/mL Tianma granules, followed by the highest concentration of BBR (50 μg/mL). Furthermore, colony formation assays demonstrated that BBR and Tianma granules noticeably suppressed the proliferative capacity of both HCT116 (Figure 8C) and SW480 cells (Figure 8D). Compared to the control group, all concentrations of BBR significantly reduced the number of colonies formed in a dose‐dependent manner by both HCT116 and SW480 cells. Consistent with the MTT assay, 1 mg/mL Tianma granules induced the most profound inhibition of colony formation, followed by 50 μg/mL BBR. Flow cytometry analysis revealed that BBR significantly promoted apoptosis in both HCT116 (Figure 8E) and SW480 cells (Figure 8F). Compared to the control group, treatment with BBR resulted in a dose‐dependent increase in the percentage of apoptotic cells in both HCT116 and SW480 cell lines. The most significant increase in apoptosis was observed with 1 mg/mL Tianma granules, followed by 50 μg/mL BBR. These results collectively indicate that berberine, similar to Tianma granules, effectively inhibits CRC cell proliferation and induces cell apoptosis in vitro, with effects generally increasing with higher concentrations of berberine, and 1 mg/mL Tianma granules showing the strongest effects among the tested treatments.

### 3.9 Berberine Inhibits CRC Cell Migration and Invasion In Vitro

**FIGURE 9:**
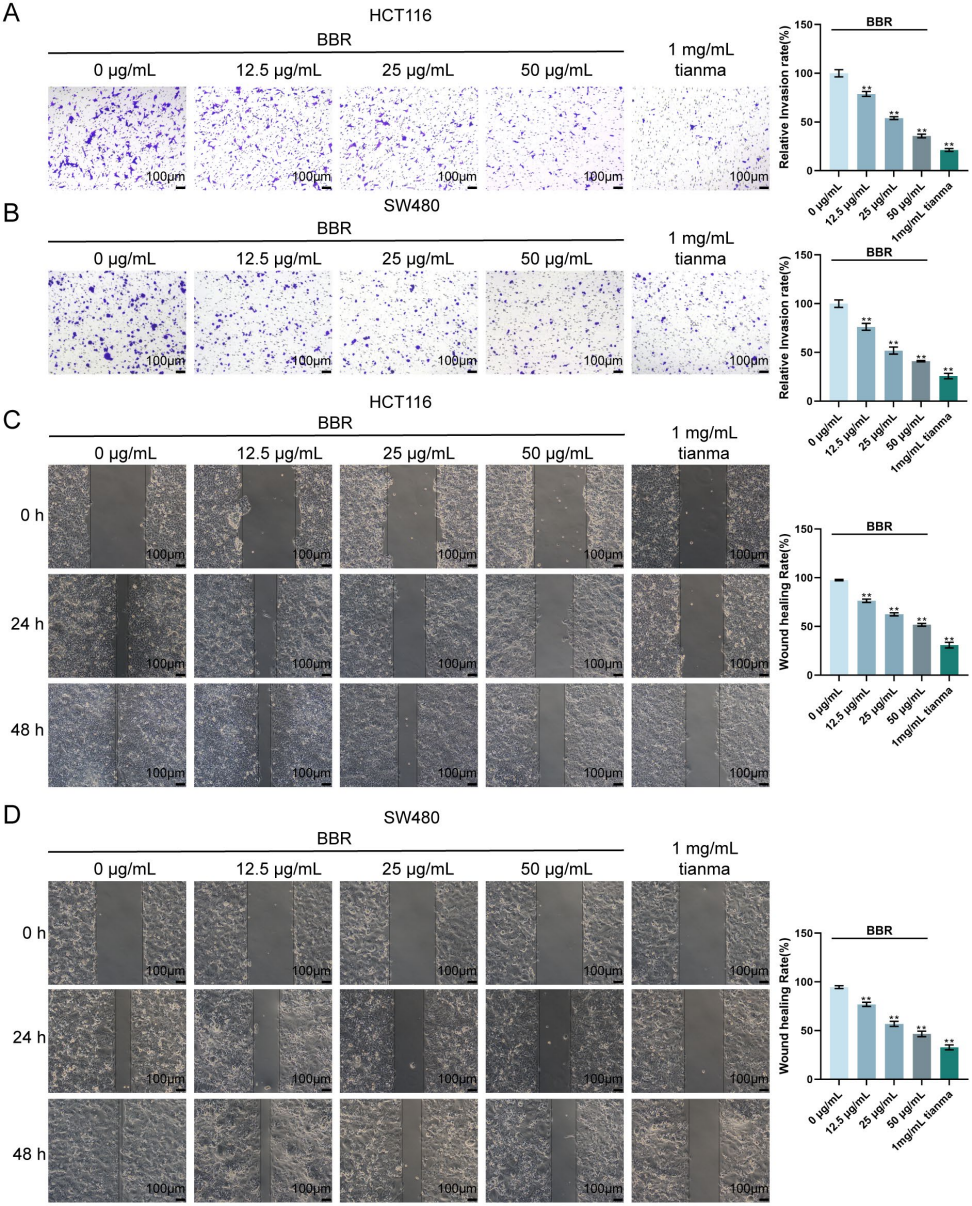
Berberine inhibits CRC cell migration and invasion in vitro. (A, B) Transwell assay results for HCT116 and SW480 cell invasion, showing representative images (left) and quantification of relative invasion rate (right). (C, D) Scratch assay results for HCT116 cell and SW480 cell migration, showing representative images at 0, 24 and 48 h (left) and quantification of wound healing rate at 48 h (right). *N* = 3. Data are expressed as mean ± SD. Statistical significance was determined by one‐way ANOVA followed by Tukey's multiple comparisons test. **p < 0.01, compared with the 0 μg/mL BBR group.

Transwell and scratch assays were performed to evaluate the effects of berberine (BBR) on CRC cell migration and invasion. Transwell invasion assays demonstrated that BBR significantly inhibited the invasion of both HCT116 (Figure 9A) and SW480 cells (Figure 9B). Compared to the untreated control group, all tested concentrations of BBR, as well as 1 mg/mL Tianma granules, resulted in a significant reduction in the relative invasion rate in a dose‐dependent manner. The most significant inhibition of cell invasion was observed with 1 mg/mL Tianma granules, followed by 50 μg/mL BBR. The scratch assay results revealed that BBR effectively reduced the migration ability of both HCT116 (Figure 9C) and SW480 cells (Figure 9D). Compared to the control group, treatment with BBR significantly decreased the wound healing rate in a dose‐dependent manner at 48 h for both cell lines. Similar to the invasion results, 1 mg/mL Tianma granules showed the most profound inhibitory effect on cell migration, followed by 50 μg/mL BBR. These findings collectively indicate that berberine effectively inhibits the migration and invasion of CRC cells in vitro, demonstrating a dose‐dependent effect, with Tianma granules showing the strongest effects among the tested treatments.

### 3.10 Berberine Promotes CRC Cell Senescence In Vitro

**FIGURE 10:**
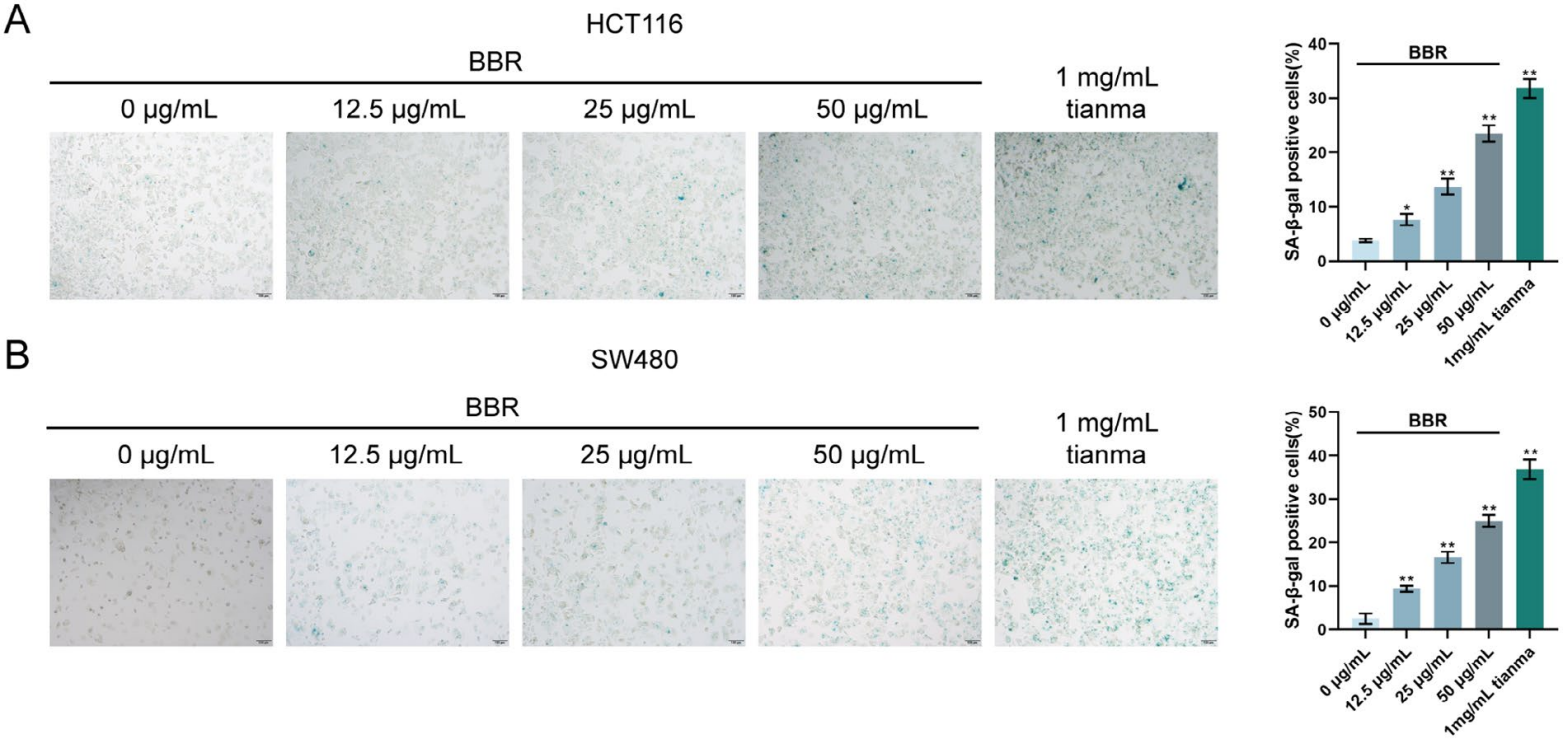
Berberine promotes CRC cell senescence in vitro. (A, B) Senescence‐associated β‐galactosidase (SA‐β‐gal) staining in HCT116 and SW480 cells, showing representative images (left) and quantification of SA‐β‐gal positive cells (right). *N* = 3. Data are expressed as mean ± SD. Statistical significance was determined by one‐way ANOVA followed by Tukey's multiple comparisons test. * *p* < 0.05, **p < 0.01, compared with the 0 μg/mL BBR group.

The induction of cellular senescence was examined using SA‐β‐gal staining to further investigate the cellular effects of berberine (BBR) on CRC cells. SA‐β‐gal staining revealed that treatment with BBR significantly increased the number of SA‐β‐gal‐positive cells in both HCT116 (Figure 10A) and SW480 cell lines (Figure 10B). Compared to the untreated control group, BBR induced a dose‐dependent increase in senescent cells. The most significant increase in SA‐β‐gal‐positive cells was observed with 1 mg/mL Tianma granules, followed by 50 μg/mL BBR. These results indicate that berberine promotes cellular senescence in CRC cells in vitro in a dose‐dependent manner, consistent with the effects observed for Tianma granules.

### 3.11 Berberine Inhibits the Activation of the Wnt/β‐Catenin Pathway in CRC Cells

**FIGURE 11:**
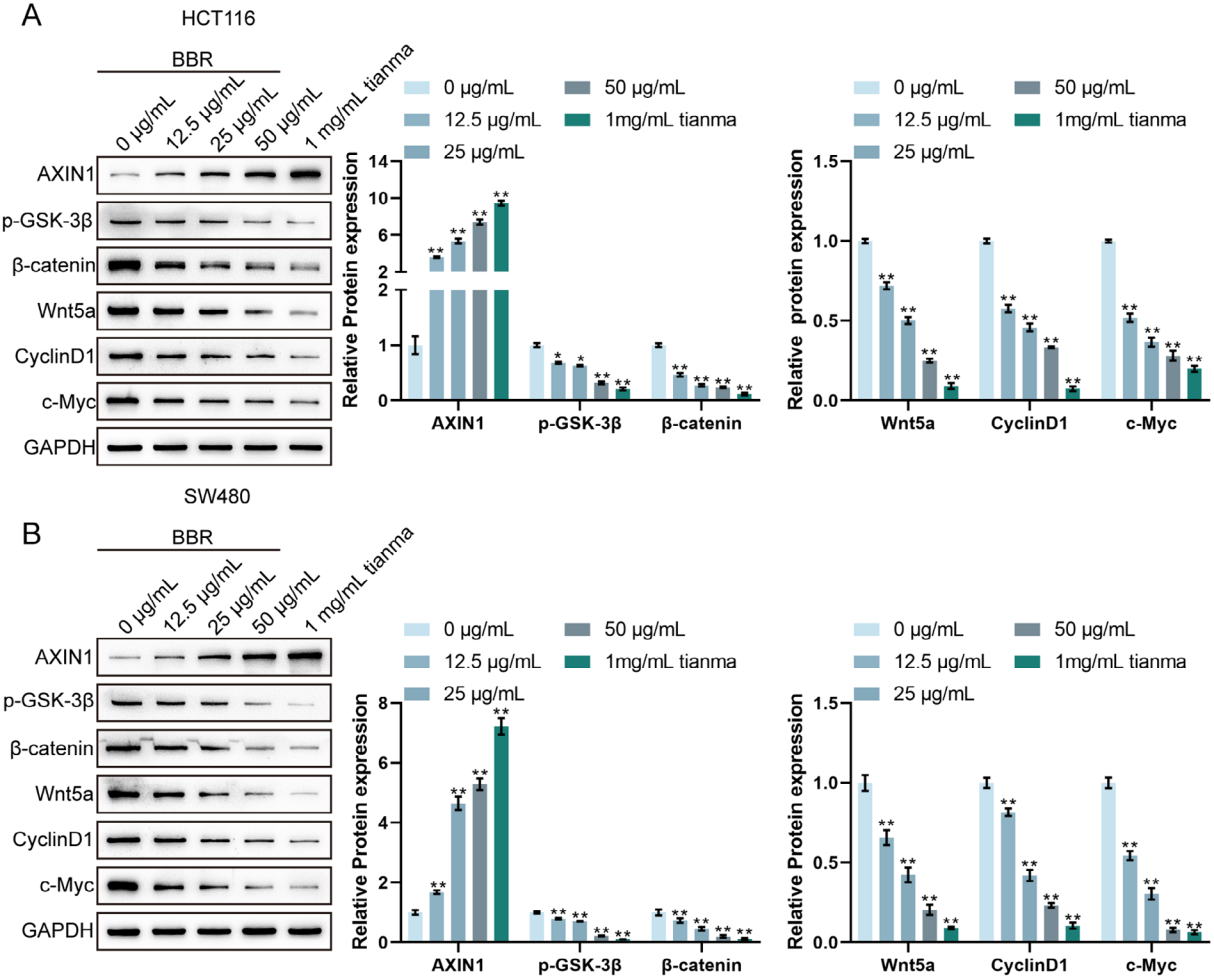
Berberine inhibits the activation of the Wnt/β‐catenin pathway in CRC cells. (A) Western blot analysis of Wnt/β‐catenin pathway‐related proteins (AXIN1, p‐GSK‐3β, β‐catenin, Wnt5a, CyclinD1 and c‐Myc) in HCT116 cells and SW480 cells, showing representative blots (left) and quantification of relative protein expression (right). GAPDH served as a loading control. *N* = 3. Data are expressed as mean ± SD. Statistical significance was determined by one‐way ANOVA followed by Turkey's multiple comparisons test. * *p* < 0.05, **p < 0.01, compared with the 0 μg/mL BBR group.

The expression levels of key proteins in the Wnt/β‐catenin signalling pathway were investigated using Western blot analysis in HCT116 and SW480 cells treated with BBR (12.5, 25 and 50 μg/mL) or 1 mg/mL Tianma granules to elucidate the molecular mechanism through which BBR exerts its anti‐cancer effects. In HCT116 cells, compared to the untreated control group, BBR treatment resulted in a dose‐dependent increase in AXIN1 protein levels and a dose‐dependent decrease in β‐catenin, Wnt5a, CyclinD1 and c‐Myc protein levels (Figure 11A). The p‐GSK‐3β also showed a dose‐dependent decrease with BBR treatment. The most significant alterations in these protein levels were observed with 1 mg/mL Tianma granules, followed by 50 μg/mL BBR. Similarly, in SW480 cells, compared to the untreated control group, BBR treatment led to a dose‐dependent increase in AXIN1 protein levels and a dose‐dependent decrease in β‐catenin, Wnt5a, CyclinD1 and c‐Myc protein levels (Figure 11B). A dose‐dependent decrease in p‐GSK‐3β levels was also observed. Consistent with the HCT116 results, 1 mg/mL Tianma granules caused the most profound changes in these protein expressions, followed by 50 μg/mL BBR. These results collectively demonstrate that berberine inhibits the activation of the Wnt/β‐catenin signalling pathway in CRC cells in a dose‐dependent manner, mirroring the effects observed with Tianma granules. This finding suggests that the Wnt/β‐catenin pathway is a crucial molecular target for the anti‐tumour activity of berberine and potentially Tianma granules in colorectal cancer.

## 4 Discussion

Inflammation is an important promoting factor in CRC progression [24, 25]. According to TCM, deficiency of spleen Qi contributes to CRC occurrence and development; spleen Qi deficiency causes water retention, which leads to stagnation of phlegm, dampness and toxicity, thereby contributing to cancerous onset. Phlegm stasis complicated by heat toxin has been recognised as a significant contributor to CRC pathogenesis [26]. TCM is characterised by its advantages of multi‐directionality, multi‐targeting, and decreased susceptibility to drug resistance. It can be used to treat tumours with multi‐factor and multi‐link pathogenesis; TCM has gained significant traction in anti‐tumour research [27]. Tianma granules intrinsically exert the activity of attacking poison and expelling evil, removing blood stasis and lumps, resolving phlegm and dissipating knots. Tianma granules combined with chemotherapy can effectively prolong the patient's survival and reduce tumour recurrence and metastasis rate [28]. For example, Tianma granules combined with capecitabine effectively prolong survival and improve advanced CRC patient quality of life [29]. Additionally, some components of Tianma granules, such as Astragalus membranaceus and seaweed, have been evidenced to possess anti‐tumour and anti‐inflammatory effects for the colon [16, 17, 18]. Therefore, Tianma granules may exhibit anti‐inflammatory and anti‐tumour effects in inflammatory colorectal cancer (CRC).

The AOM/DSS model is widely used for CRC studies in animals. As evidenced previously, albuca bracteate polysaccharides could attenuate AOM/DSS‐induced colon tumourigenesis by reducing colon shortening, weight loss and histological damage in CRC mice [30]. Evodiamine can significantly reduce the number and size of AOM/DSS‐induced CRC tumours, while promoting cell apoptosis and alleviating intestinal inflammation [31]. Chang‐wei‐qing decoction improves AOM/DSS‐induced colon length decrease, reduces tumour count and improves colitis score [32]. In this study, Tianma granules could improve AOM/DSS‐induced decrease in mouse body weight, survival rate and colon length, and reduce CRC tumour numbers. Based on previous literature [30, 31, 32], Tianma granules can inhibit AOM/DSS‐induced CRC progression. Moreover, the oral toxicity evaluation demonstrated that Tianma granules administered at a clinically relevant dose produced no significant alterations in weight, liver/renal functions and histology of major organs. This evidence supports the safety profile of this formula.

After confirming the anti‐tumour effect of Tianma granules, the influence of Tianma granules in colon tissue inflammation in CRC mice was further analysed. The results showed that Tianma granules reduced inflammatory cell infiltration and tumour numbers in the colon, increased goblet cell numbers, and promoted CRC cell apoptosis in CRC mice models. Additionally, Tianma granules decreased TNF‐α, IL‐1β and IL‐6 levels and increased IFN‐γ levels; Tianma granules also decreased COX2, MUC2 and Bcl2 protein levels, while elevating Bax and Cleaved caspase3 levels. A previous study has shown that goblet cells play a crucial role in maintaining and protecting the intestinal barrier [33]. In IBD, goblet cell defects can lead to the occurrence of advanced cancer [34]. TNF‐α, IL‐1β and IL‐6 are pro‐inflammatory cytokines, and their upregulation indicates inflammation, which can induce CRC [35]. COX2 and MUC2 are specific biomarkers for CRC [36], which are highly expressed in CRC. Additionally, colon epithelial cells may undergo cell death after inflammation and DNA damage, which may inhibit the development of CRC tumours [37]. Dysregulated apoptosis of colon epithelial cells ultimately leads to CRC progression [38, 39]. Similarly, it has been demonstrated that increased tumour cell apoptosis is closely associated with CRC treatment [31]. Tianma granules alleviated AOM/DSS‐induced inflammation and cellular carcinogenesis in CRC.

It has been suggested that tumours develop as a result of an imbalance between cell proliferation and apoptosis [40]. A key characteristic of tumours is metastasis, specifically the migration and invasion of tumour cells [41]. The regulatory effect of Tianma granules on the biological function of CRC cells was further observed in vitro in this study. After Tianma granule treatment, HCT116 cells and SW480 cells showed decreased cell viability and proliferation ability, and increased apoptosis rate; Tianma granules treatment increased Bax and cleaved caspase3 levels, decreased Bcl2 level, as well as inhibited CRC cell migration and invasion ability. Consistently, it has been shown that baicalin can inhibit CRC cell proliferation and migration, promote CRC cell apoptosis, elevate Bax protein level, and inhibit Bcl2 level inhibition [42]. Moreover, cellular senescence, defined as an irreversible cell cycle arrest caused by various internal and external insults [43], has traditionally been considered a defence mechanism against cancer progression [44]. Pro‐senescence therapies help limit the damage by restraining proliferation and fibrosis during carcinogenesis and active tissue repair, whereas anti‐senescence agents enable the elimination of accumulated senescent cells to restore tissue function, and potentially be conducive to organ rejuvenation [45, 46]. In this study, Tianma granules promoted cellular senescence and induced cell cycle arrest, as evidenced by a significant increase in SA‐β‐gal‐positive cells and an accumulation of cells in the G1 phase, along with decreased levels of CDK4, CDK6 and Lamin B1 [47]. These findings highlight the potential of Tianma granules to inhibit the proliferation, migration, and invasion of CRC cells, while inducing apoptosis, cellular senescence and cell cycle arrest.

The Wnt signalling cascade plays an indispensable role in various biological processes, including embryonic development, cell cycle regulation, inflammation and cancer [48]. The Wnt pathway‐related components have been evidenced to be reliable biomarkers and potential targets for cancer treatment [49, 50]. As revealed previously, baicalin improves AOM/DSS‐induced CRC by inhibiting the Wnt/β‐Catenin signalling cascade [51]. Cinobufacini can inhibit the invasion and metastasis of CRC, which is mediated by inhibiting the Wnt/β‐catenin signalling pathway [52]. In this study, Tianma granules‐treated CRC mice and cells showed increased AXIN1 levels and decreased β‐catenin, Wnt5a and CyclinD1 levels. It has been shown that increased AXIN1 levels and decreased β‐Catenin, Wnt5a and CyclinD1 levels indicate the inhibition of the Wnt/β‐catenin pathway [53]. Taken together, the Wnt/β‐catenin signalling pathway was activated in CRC mice and cells, which could be inhibited by Tianma granules.

A network pharmacology approach was employed to investigate the specific active components and underlying mechanisms of Tianma granules in greater detail. This analysis predicted key active compounds and their targets within Tianma granules, with a particular focus on the Wnt signalling pathway, which is known to be critically involved in colorectal cancer (CRC) pathogenesis [54]. Our in vitro validation experiments then confirmed that berberine, a prominent compound of Tianma [55] identified by network pharmacology, significantly inhibited CRC cell proliferation, migration and invasion, and induced apoptosis and senescence. Previous studies have similarly reported that compounds like baicalin can inhibit CRC cell proliferation and migration and promote apoptosis [56]. Regarding berberine, it has been reported to inhibit the proliferation, migration and invasion of CRC cells [57, 58], further underscoring the potential of natural compounds in CRC therapy. Crucially, berberine also modulated the Wnt/β‐catenin signalling pathway by increasing AXIN1 and decreasing β‐catenin, Wnt5a, CyclinD1 and c‐Myc protein levels, alongside the reduction of p‐GSK‐3β. Previous studies demonstrated that inhibition of the Wnt/β‐catenin pathway is beneficial for CRC treatment [59] and that compounds such as cinobufacini can inhibit CRC progression by suppressing this pathway [60]. More importantly, berberine and its synthetic derivatives represent potential therapeutic agents to inhibit Wnt/β‐catenin signalling in tumorigenesis [61]. Therefore, the present findings strongly suggest that berberine is a significant active component contributing to the therapeutic effects of Tianma granules against CRC, primarily through its modulation of the Wnt/β‐catenin pathway. While Tianma granules are a complex herbal prescription with multiple active compounds, the elucidation of berberine's role marks a significant step towards understanding the multi‐target mechanisms of this traditional medicine.

This study provides compelling evidence for the anti‐colorectal cancer effects of Tianma granules and elucidates its mechanism through the Wnt/β‐catenin pathway. Nevertheless, certain limitations should be acknowledged. While our network pharmacology analysis provided valuable insights, a comprehensive omics approach (e.g., proteomics) is needed for a more exhaustive understanding of global molecular changes and novel targets. Future research should also aim to fully characterise other active compounds, investigate their synergistic effects, assess potential drug‐herb interactions, assess long‐term safety, and explore their clinical translation. In addition, pharmacokinetic (PK) characterisation is fundamental for drug development. Current PK data on Tianma granules remain lacking. Notably, berberine exhibits poor oral bioavailability due to extensive first‐pass metabolism [62], highlighting the need for innovative delivery strategies or structural modifications to enhance its therapeutic potential.

In summary, this study demonstrated that Tianma granules can inhibit inflammation and tumour development in AOM/DSS‐induced CRC mice. Moreover, Tianma granules can also inhibit CRC cell proliferation, migration and invasion, and induce apoptosis in vitro. The protection of Tianma granules against CRC may be mediated by inhibition of the Wnt signalling pathway, and berberine might be the key compound responsible for Tianma granules' effects. Our study demonstrated that Tianma granules are promising protective agents for inflammatory colorectal cancer (CRC). However, further experiments are needed to fully comprehend the underlying mechanisms.

## Author Contributions

**Renjing Lin:** conceptualization (equal), data curation (equal), formal analysis (equal), investigation (lead), software (equal), validation (lead), visualization (lead), writing – original draft (lead). **Jinyin Xiao:** data curation (equal), formal analysis (equal), investigation (equal), software (equal), validation (equal), visualization (equal). **Yanjie Chen:** data curation (equal), investigation (equal), validation (equal), visualization (equal). **Xu Li:** data curation (equal), investigation (equal), validation (equal). **Peiwen Zhang:** software (equal), validation (equal), visualization (equal). **Runcheng Zhang:** investigation (equal), validation (equal), visualization (equal). **Min Luo:** conceptualization (lead), data curation (lead), formal analysis (lead), funding acquisition (lead), methodology (lead), project administration (lead), resources (lead), supervision (lead), writing – review and editing (lead).

## Ethics Statement

All animal experiments were approved by the Animal Experiment Ethics Committee of Hunan University of Chinese Medicine.

## Conflicts of Interest

The authors declare no conflicts of interest.

## Supporting information


**Figure S1:** HPLC‐MS of Tianma granule. (A) Positive ion mode of Tianma granule; (B) negative ion mode of Tianma granule; (C) positive ion mode of reference standards; (D) negative ion mode of reference standards. a: phellodendrine; b: astragaloside A; c: rheic acid; d: chrysophanol.

**Figure S2:** The oral toxicity of Tianma granules. Six C57BL/6 mice were orally administered high‐dose Tianma granule once per day for 8 weeks. (A) Body weight. (B–F) The histological analysis of heart, liver, spleen, lung and kidney. (G) The serum level of ALT, AST, Cr and BUN.

## Data Availability

The data supporting this study's findings are available from the corresponding author upon reasonable request.
