# Supplementary figures and images for: Tianma Granules Alleviate AOM/DSS‐Induced Colorectal Tumorigenesis by Inhibiting the Wnt/β‐Catenin Pathway Activation
Source: J Cell Mol Med. 2025 Aug 10;29(15):e70772. doi: 10.1111/jcmm.70772 (PMC12336053; doi:10.1111/jcmm.70772)

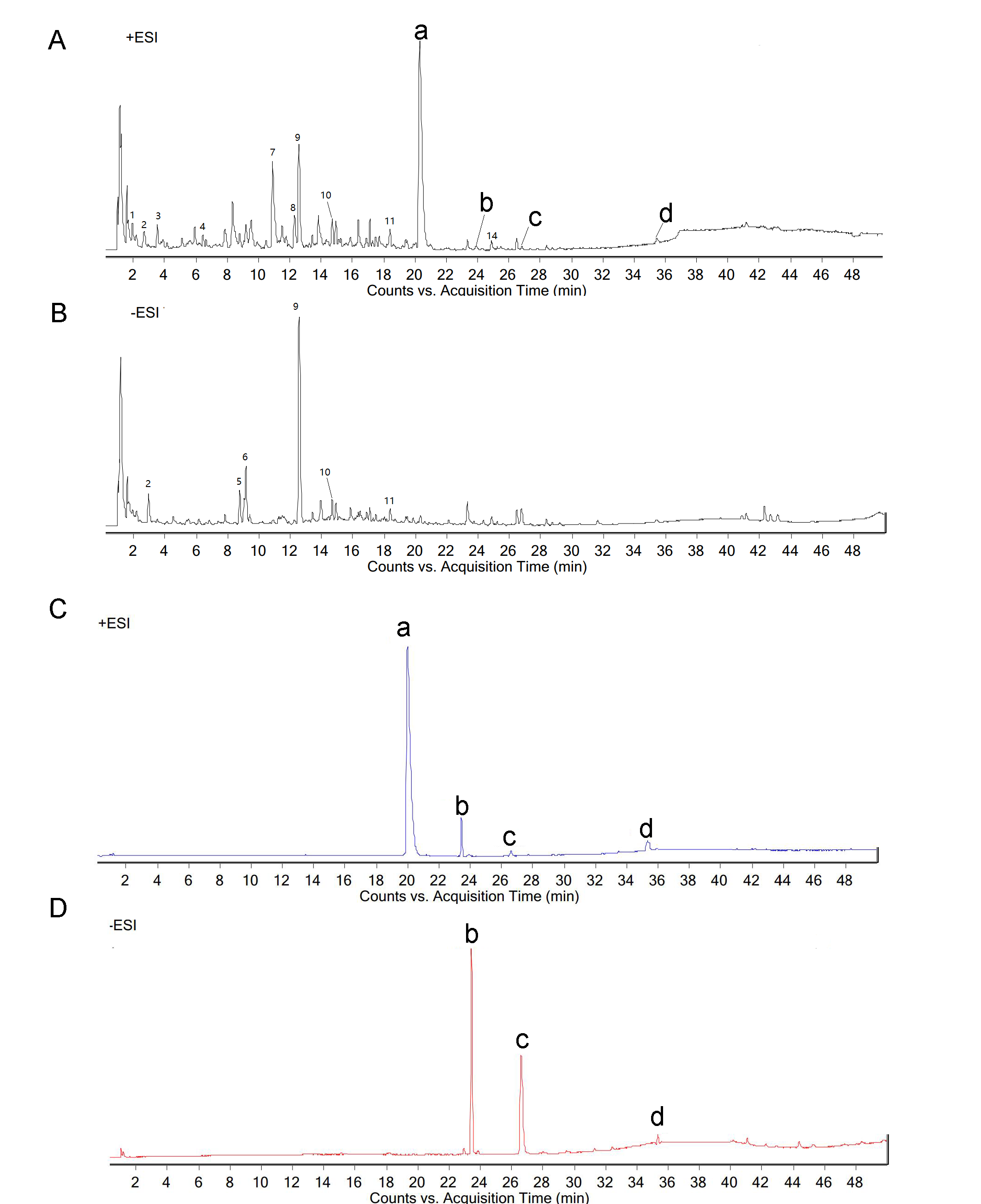

Supplement: Supplementary file 1 — Figure S1: HPLC‐MS of Tianma granule. (A) Positive ion mode of Tianma granule; (B) negative ion mode of Tianma granule; (C) positive ion mode of reference standards; (D) negative ion mode of reference standards. a: phellodendrine; b: astragaloside A; c: rheic acid; d: chrysophanol. [file JCMM-29-e70772-s001.tif]

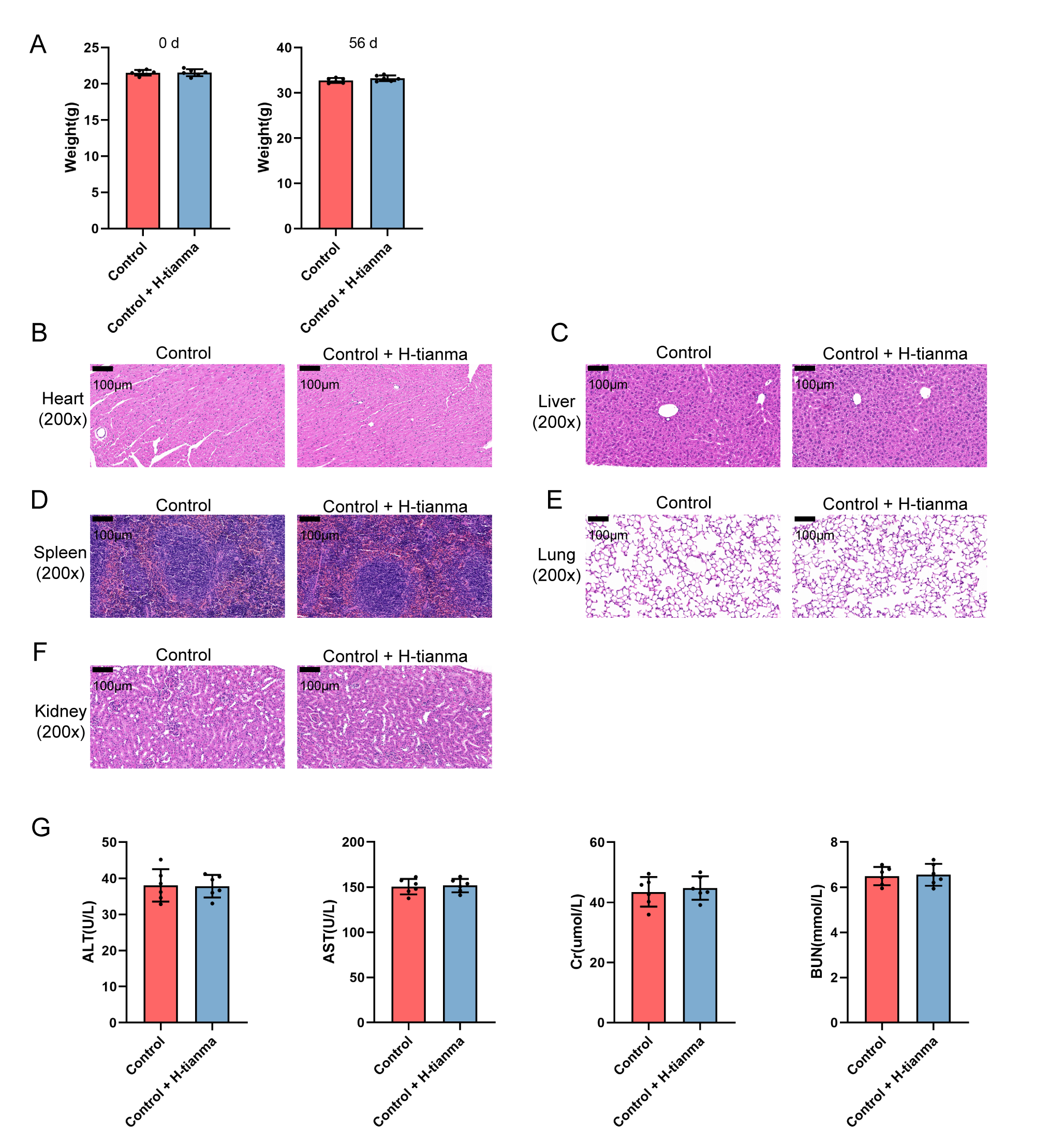

Supplement: Supplementary file 2 — Figure S2: The oral toxicity of Tianma granules. Six C57BL/6 mice were orally administered high‐dose Tianma granule once per day for 8 weeks. (A) Body weight. (B–F) The histological analysis of heart, liver, spleen, lung and kidney. (G) The serum level of ALT, AST, Cr and BUN. [file JCMM-29-e70772-s002.tif]
